# EIF4A3-induced Circ_0001187 facilitates AML suppression through promoting ubiquitin-proteasomal degradation of METTL3 and decreasing m6A modification level mediated by miR-499a-5p/RNF113A pathway

**DOI:** 10.1186/s40364-023-00495-4

**Published:** 2023-06-06

**Authors:** Xinyu Yang, Fengjiao Han, Xiang Hu, Guosheng Li, Hanyang Wu, Can Can, Yihong Wei, Jinting Liu, Ruiqing Wang, Wenbo Jia, Chunyan ji, Daoxin Ma

**Affiliations:** 1 Department of Hematology, Qilu Hospital of Shandong University, Cheeloo College of Medicine, Shandong University, Jinan, Shandong 250012 People’s Republic of China; 2 Shandong Provincial Key Laboratory of Immunohematology, Qilu Hospital of Shandong University, Jinan, Shandong People’s Republic of China

**Keywords:** Circ_0001187, METTL3, RNF113A, miR-499a-5p, Acute myeloid leukemia, Progression

## Abstract

Aberrant expression of circRNAs has been proven to play a crucial role in the progression of acute myeloid leukemia (AML); however, its regulatory mechanism remains unclear. Herein, we identified a novel circRNA, Circ_0001187, which is downregulated in AML patients, and its low level contributes to a poor prognosis. We further validated their expression in large-scale samples and found that only the expression of Circ_0001187 was significantly decreased in newly diagnosed (ND) AML patients and increased in patients with hematological complete remission (HCR) compared with controls. Knockdown of Circ_0001187 significantly promoted proliferation and inhibited apoptosis of AML cells in vitro and in vivo, whereas overexpression of Circ _0001187 exerted the opposite effects. Interestingly, we found that Circ_0001187 decreases mRNA m^6^A modification in AML cells by enhancing METTL3 protein degradation. Mechanistically, Circ_0001187 sponges miR-499a-5p to enhance the expression of E3 ubiquitin ligase RNF113A, which mediates METTL3 ubiquitin/proteasome-dependent degradation via K48-linked polyubiquitin chains. Moreover, we found that the low expression of Circ _0001187 is regulated by promoter DNA methylation and histone acetylation. Collectively, our findings highlight the potential clinical implications of Circ _0001187 as a key tumor suppressor in AML via the miR-499a-5p/RNF113A/METTL3 pathway.

**Supplementary Information:**

The online version contains supplementary material available at 10.1186/s40364-023-00495-4.

## Key points

1. Circ_0001187 is downregulated in AML patients and contributes to poor prognosis.
2. Circ_0001187 suppresses AML progression by sponging miR-499a-5p to promote RNF113A-mediated METTL3 ubiquitination.

**Supplementary Information:**

The online version contains supplementary material available at 10.1186/s40364-023-00495-4.

## Introduction

Although great advances have been made in acute myeloid leukemia (AML) therapy, many patients still experience disease progression and poor outcomes after conventional and targeted therapies or methods [1, 2]. Further investigation is critical to fully understand the molecular mechanisms underlying AML progression.

CircRNAs, a novel class of covalently closed RNAs, play important roles in carcinogenesis, have been proven to be involved in resistance to treatment, and are expected to be cancer therapeutic targets [3, 4]. Accumulating evidence has demonstrated that circRNAs act as important epigenetic regulators of AML progression and are expected to be therapeutic targets. It has been reported that circMYBL2 regulates FLT3 kinase activity and promotes FLT3-ITD leukemia progression [5]. Our previous studies have also clarified the expression profile of circRNAs in cytogenetically normal AML patients and demonstrated that hsa_circ_0004277 and hsa_circ_0001947 are essential modulators of AML progression [6, 7]. Therefore, considering the role of circRNAs in AML pathogenesis, further studies are needed to uncover the underlying molecular mechanisms.

N6-methyladenosine (m^6^A) has been proven to influences RNA functions and plays crucial roles in hematopoietic stem cells (HSCs) biology and leukemic transformation [8–13]. METTL3, the core methyltransferase involved in m^6^A modification, has been reported to play significant roles in normal hematopoiesis and hematological malignancies including AML, acute lymphocytic leukemia (ALL), chronic myeloid leukemia (CML), and lymphoma [14, 15]. Elevated expression of METTL3 in AML has been shown to promote proliferation and chemoresistance, and decrease apoptosis in leukemia cells [14]. Further studies on the regulatory mechanisms of METTL3 in hematological diseases are required. Therefore, exploring additional therapeutic targets, revealing their relationship with the disease, and providing a foundation for developing novel inhibitors would facilitate the development of a potentially targeted METTL3 therapy in clinical practice.

In this study, we identified a novel circRNA, Circ_0001187, and further confirmed that it functions as a tumor suppressor in AML and that its lower level contributes to poor prognosis. We further explored the epigenetic regulation mechanisms underlying Circ_0001187 silencing and post-transcriptional biogenesis mediated by the RNA-binding protein EIF4A3. Functional studies demonstrated that Circ_0001187 suppresses proliferation and promotes apoptosis of AML cells by destabilizing METTL3 via a competing endogenous RNA (ceRNA) mechanism to sponge miR-499a-5p and upregulate the E3 ligase RNF113A. Our findings have defined an important tumor-suppressive circRNA in AML and provided a potential strategy to induce METTL3 degradation in leukemia treatment.

## Materials and methods

### Patients and samples collection

BM samples from newly diagnosed (ND) non-M3 AML patients, relapsed-refractory (R/R) patients, hematological complete remission (HCR) patients, and controls were obtained following informed consent at the Qilu Hospital of Shandong University. AML patients were diagnosed according to the French-American-British (FAB) classification system. Their characteristics are summarized in Table 1, and the risk stratification standards are shown in Supplementary Table 1. Hematological complete response (HCR) was defined based on the International Working Group Criteria [16]. The exclusion criteria were as follows: age < 16 years and > 70 years, receiving antibiotics or hormone therapy within the last 10 weeks, and blood pressure anomalies. The study was conducted in accordance with the Declaration of Helsinki and the protocol was approved by the Ethics Committee of Qilu Hospital.

**Table 1:** Clinical and laboratory characteristics of the newly diagnosed (ND) AML patients

**Characteristics**	**No. /treatments**
Male/female	31/39
Age at study entry, year, median (range)	44(13-83)
Bone marrow blasts at diagnosis, %, median ( range)	85.5(28-98)
Peripheral Blood blasts at diagnosis, %, median ( range)	84.5(21-98)
WBC at diagnosis, ×10^9^/L, median (median range)	16.13(1.21-89.17)
PLT at diagnosis, ×10^9^/L, median (median range)	34.5(6-191)
HGB at diagnosis, g/L, median (median range)	81(51-135)
AML with minimal differentiation: M0	0
AML without maturation: M1	2
AML with maturation: M2	9
Acute Promyelocytic leukemia: M3	0
Acute Myelomonocytic leukemia: M4	23
Acute monoblastic or monocytic leukemia: M5	36
Acute erythroid leukemia: M6	0
Acute megakaryoblastic leukemia: M7	0
Preferred treatments	DA/IA (3+7)

*WBC* white blood cell, *PLT* platelet, *HGB* hemoglobin, *DA* Daunorubicin+Cytarabine, *IA* idarubicin+Cytarabine

### Antibodies and reagents

The primary antibodies against METTL3 (#86132), AGO2 (#82897), Histone H3K9ac (#9649), Histone H4K5ac (#8647) were purchased from Cell Signaling Technology (Beverly, MA, USA), and antibodies against RNF113A (#27018-1-AP), EIF4A3 (#17504-1-AP), MDM2 (#66511-1-lg), P21 (#10355-1-AP), P53 (#10442-1-AP), HA-Tag (#66006-2-Ig), Flag (#66008-4-lg, #20543-1-AP) were obtained from proteintech (USA). Antibody against GAPDH (ab8245) was obtained from Abcam (Cambridge, UK). HRP-conjugated secondary antibodies, goat-anti-mouse-HRP (AB_2338504) and goat anti-rabbit-HRP (AB_2337938), were bought from Jackson ImmunoResearch. The METTL3 inhibitor STM2457, Chidamide, 5-Azacytidine, Ara-C, and MG132 were purchased from MCE (USA).

### Cell culture

AML cell lines (THP-1 and Molm-13) and 293 T cells were obtained from the Cell Bank of the Type Culture of the Chinese Academy of Sciences (Shanghai, China). THP-1 cells were cultured in RPMI-1640 (Gibco, NE, USA) supplemented with 10% fetal bovine serum (FBS; Biological Industries, Beit HaEmek, Israel). Molm-13 cells were cultured in IMDM (Gibco, NE, USA) supplemented with 20% FBS and 293 T cells were cultured in Dulbecco’s modified Eagle’s medium (DMEM; Gibco, NE, USA) supplemented with 10% FBS. All the cells were cultured in a humidified atmosphere at 37◦C and 5% CO_2_.

### Quantitative reverse transcription PCR (qRT-PCR)

Total RNA was isolated using TRIzol (Ambion, CA, USA), then reversely transcribed into cDNA at 37 °C for 15 min followed by 85 °C for 10 s using M-MLV RTase cDNA Synthesis Kit (Takara Bio Inc., Japan). Quantitative PCR (qPCR) was performed using a Roche Applied Science Light Cycler 480II Real-time PCR system (Roche Applied Science, IN, USA) in accordance with the manufacturer’s instructions. The AceQ qPCR SYBR Green Master Mix (Takara, Japan) was used for qPCR. The PCR mixture contained 5 μL of 2 × SYBR Green Real-time PCR Master Mix, 0.5 μL of the forward and reverse primers, 1 μL of cDNA, and 3 μL ddH_2_O, in a final volume of 10 μL. PCR conditions were performed as follows: 95 °C for 10 min, followed by 40 cycles (95 °C for 20 s and 60 °C for 1 min). Primer sequences for the relevant genes are listed in Supplementary Table 2. Gene expression was expressed relative to the endogenous control GAPDH and calculated using the 2 ^–△CT^ method.

### RNase R treatment

To assess the stability of Circ _0001187 and its linear DOPEY2 mRNA, we first digested total RNA (1 μg) of THP-1 with 4 U RNase R at 37◦C for 1 h. The expression levels of these two genes were determined using qRT-PCR and agarose gel electrophoresis.

### Lentivirus, siRNA, plasmid construction, and cell transfection

AML cells were infected with lentivirus containing short hairpin RNA (shRNA) of Circ _0001187 or lentivirus overexpressing Circ_0001187. A fluorescence microscope was used to determine infection efficiency after 48 h, and puromycin was used to obtain stably infected cells. The qRT-PCR was used to detect the mRNA expression changes in Circ_0001187 after lentivirus infection in AML cells.

The siRNAs of Circ_0001187, EIF4A3, RNF113A, METTL3, TRIM21, TRIM33, UBR5, MYCBP2, TRIM56, TRIM41, HUWE1, or HERC2 and their related control oligonucleotides were precisely designed and produced by Gene Pharma (Shanghai, China). The miR-499a-5p mimics or inhibitor and related control oligonucleotides were also precisely designed and provided by Gene Pharma (Shanghai, China). FLAG-tagged expression vectors for full-length METTL3 and GFP-tagged expression vectors for full-length RNF113A and HA-UB plasmids were obtained from Boshang (Jinan, China). For siRNA and plasmid transfection, cells were transfected using ExFect 2000 Transfection Reagent (Vazyme, China) according to the manufacturer’s instructions. All sequences are listed in Supplementary Table 3.

### Cell proliferation assays

After the different treatments, the cells were seeded in 96-well plates and incubated with 10μL of CCK-8 (Beyotime, China) for 3 h. The absorbance was then measured at 450 nm. All experiments were performed in triplicate.

### Apoptosis assay

Apoptosis was detected using an Annexin V-FITC/PI apoptosis detection kit (BestBio, Shanghai, China), according to the manufacturer’s instructions. Briefly, after different treatments, the cells were resuspended in 400μL of binding buffer and stained with 5μL of Annexin V for 15 min, followed by 10μL of PI for another 5 min. The stained cells were analyzed using a Beckman Gallios cytometer. The final test results were subsequently analyzed using Kaluza software (Beckman Coulter, WI, USA).

### Fluorescence In situ hybridization (FISH)

FISH was performed using a probe specific to Circ_0001187 or miR-499a-5p sequences, which were purchased from Gene Pharma (Shanghai, China). Hybridization was performed according to the manufacturer’s instructions using a FISH Kit (Gene Pharma, Shanghai, China). The probe sequences: Circ_0001187 antisense probe: CTCTTGGAGGTTGTGTTTGGTGGTT; Circ_0001187 sense probe: AACCACCAAACACAACCTCCAAGAG; miR-499a-5p antisense probe: AAACATCACTGCAAGTCTTAA; miR-499a-5p sense probe: TTAAGACTTGCAGTGATGTTT. All images were viewed using a Laser Confocal Microscope (Carl ZEISS, LSM900, Germany).

### Immunoprecipitation (IP) assay

Cells were lysed in IP buffer containing a protease inhibitor and phosphatase inhibitor cocktail (Beyotime, China) for 20 min on ice. The lysates were centrifuged at 12,000 g for 15 min, and the supernatant was incubated with antibody-binding magnetic beads at 4 °C overnight. A sample buffer of 1 × SDS was added to the magnetic beads and the immunoprecipitated products were used for western blot analysis.

### Western blot analysis

The cells were lysed with RIPA buffer (Beyotime, China) containing a protease inhibitor (Beyotime, China) on ice. A bicinchoninic acid (BCA) protein assay kit (Beyotime, China) was used to measure protein concentrations. Protein extracts (30 μg) or IP products were separated by SDS-PAGE and transferred to nitrocellulose membranes (Millipore, Bedford, MA, USA). After overnight with specific primary antibodies at 4 °C, the membranes were incubated with HRP-conjugated secondary antibodies at room temperature for 1 h. Next, we detected the protein bands after washing.

### RNA Pull-Down and miRNA sequencing

THP-1 cells (8 × 10^7^) were lysed and incubated with a biotin-conjugated probe for Circ_0001187. The Circ_0001187 probe and negative control probe were synthesized by Cloud-Seq (Shanghai, China). Streptavidin-binding magnetic beads were added to the mixture and incubated for 12 h. The target RNA bound to the beads was separated using a biotin probe and subjected to high-throughput miRNA sequencing (Cloud-Seq; Shanghai, China). The probe sequences: Circ_0001187 sense probe: TTGGAGGTTGTGTTTGGTGGTTTTA; Circ_0001187 antisense probe: TAAAACCACCAAACACAACCTCCAA.

### Actinomycin D assay

Cells were exposed to actinomycin D (10 μg/mL) for the indicated times (0, 4, 8, and 12 h), and total RNA was isolated using TRIzol. The expression of Circ_0001187 was determined by qRT-PCR.

### Methylation-specific PCR (MSP)

Genomic DNA was extracted according to the manufacturer’s protocol (TIANGEN Biotech Code No.: DP304-02). The DNA samples were first treated with the EpiArt® DNA Methylation Bisulfite Kit (Vazyme, Code No: EM101-01) and then amplified by PCR (Vazyme, Code No: EM202-01). The methylation status of Circ_0001187 was determined using MSP with primers specific for the methylated and unmethylated alleles of each gene after treatment of the genomic DNA with sodium bisulfite. Detailed primer sequences are summarized in Supplementary Table 4. For MSP, the PCR products were analyzed on 2% agarose gels, stained with ethidium bromide, and visualized under UV light.

### Dual luciferase reporter assay

Mut-hsa-miR-499a-5p fragment was synthesized and inserted downstream of the luciferase reporter gene of the pmirGLO Vector (Gene Pharma, China) and a wild-type was a negative control for this experiment and then transfected into 293T cells using ExFect 2000 Transfection Reagent (Vazyme, China). Next, we transfected the cells with hsa-miR-499a-5p mimics or a negative control. After 48 h, the Dual Luciferase Reporter Assay System (Promega) was used to detect firefly and Renilla luciferase activity by microplate reader (BioTek, VT, USA). Finally, we used the ratio of Read1:Read2 to represent the fluorescence activity.

### RNA immunoprecipitation (RIP) assay

A Magna RIP RNA-binding Protein Immunoprecipitation Kit (Millipore, Billerica, MA, USA) was used to perform RIP experiments according to the manufacturer’s instructions. Briefly, THP-1 cells (1 × 10^7^) were lysed in 1 mL RIP lysis buffer containing RNase inhibitors. The cell lysates were incubated with beads coated with IgG or anti-AGO2 antibodies on a rotator at 4 °C overnight. Total RNA was extracted with an RNeasy MinElute Cleanup Kit (Qiagen, Valencia, CA, USA) and the DOPEY2 promoter was detected by qRT-PCR.

### Chromatin immunoprecipitation (ChIP)

ChIP analysis was performed using a ChIP Assay Kit (Millipore) following the manufacturer’s instructions. The cells were subjected to ChIP analysis using antibodies against acetyl-histone H3 (Lys9) and acetyl-histone H4 (Lys5) at 4 °C overnight. The enriched DNA associated with the promoter region of Circ_0001187 was quantified by qPCR (see Supplementary Table 4 for primer sequences).

### Immunofluorescence (IF)

THP-1 and Molm-13 cell suspensions (2 × 10^5^) were dropped onto polylysine-treated slides. The cells were fixed with 4% paraformaldehyde for 20 min and treated with 0.1% Triton X-100 in PBS for 10 min. Cells were blocked with 5% BSA for 30 min at 37 °C, incubated with primary antibody overnight at 4 °C, and washed with PBS three times. The cells were incubated with DAPI for 30 min. All images were viewed using a Laser Confocal Microscope (Carl ZEISS, LSM900, Germany).

### m^6^A dot blot

The mRNA samples were denatured by heating at 72 °C for 5 min, followed by immediate chilling on ice. Then, mRNA was dropped onto the Amersham Hybond™-N + membrane (GE Healthcare, #RPN303B) and crosslinked to the membrane twice by UV in autocross link mode (1,200 microjoules [× 100]; 25–50 s) using UVP Crosslinker Analytik-jena. One membrane was blocked and detected using an m^6^A-specific antibody (Synaptic Systems). The other membrane was stained with methylene blue and used as a loading control.

### AML murine models

Six-week-old male NOD-Prkdcscid-Il2rgem1IDMO (NPI) mice were obtained from Beijing IDMO Co. Ltd. (Beijing, China) and randomly divided into two groups. They were injected with 5 × 10^6^ THP-1 cells transfected with sh-Circ_0001187-GFP or Ctrl-GFP via the tail vein to establish a murine AML model. AML mice were sacrificed after 40 days, and the leukemia burden and complications in the spleen, liver, and bone marrow were analyzed.

### Statistical analysis

The data were analyzed using GraphPad Prism 8.0 and SPSS 25 software (IBM, USA). We first analyzed the data using the F-test (homogeneity test of variance) and followed the methods of t-test analysis for the data of the two groups for comparison. Student’s t-test and two-way ANOVA were used to assess differences in variables between the groups. And corrected *P*-value were analyzed using Bonferroni. Specific statistical methods are described in the corresponding illustrations of the results. Statistical significance was set at *P* < 0.05. ns, no significance; *p < 0.05; **p < 0.01; ***p < 0.001; ****p < 0.0001.

## Results

### Circ_0001187 is decreased in AML patients and its low level correlates with poor prognosis

**Fig. 1:**
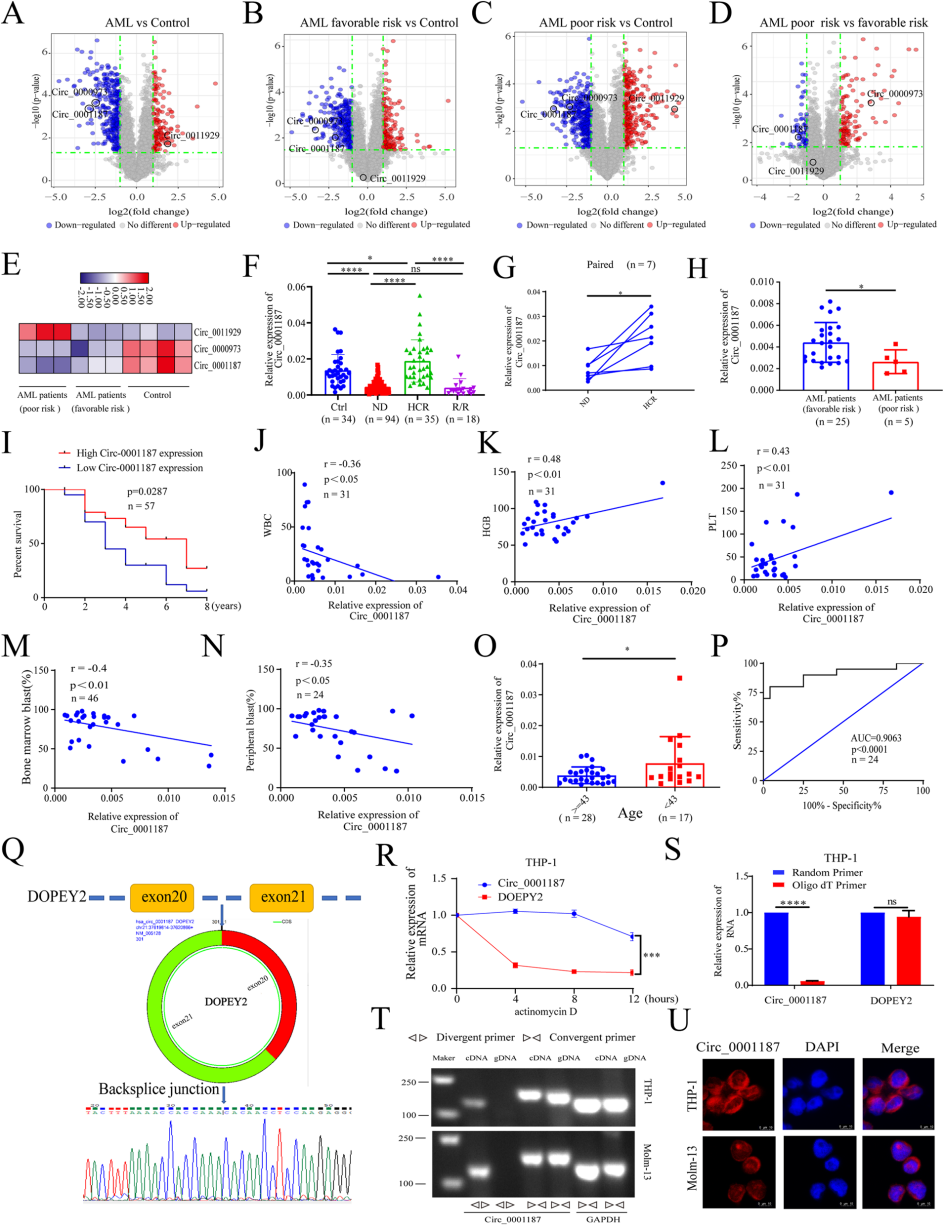
Decreased Circ_0001187 in AML correlates with poor prognosis and characteristics of Circ_0001187 in AML cells. **A**-**D** Volcano plots of differentially expressed circRNAs between AML patients (favorable risk and poor risk) and healthy controls. **E** Heatmap of differentially expressed circRNAs between AML patients and healthy controls (blue = down-regulated, red = up-regulated). Fold change was listed above. **F** The expressions of Circ_0001187 in AML patients and healthy controls. **G** Circ_0001187 mRNA expression was measured in paired samples from five follow-up AML patients at ND and HCR stages. Data were analyzed using Wilcoxon test. **H** qRT-PCR analysis of Circ_0001187 expression in AML patients with favorable risk and poor risk. **I** Kaplan–Meier analysis of the overall survival time of AML patients with different circRNAs expression as indicated by Log-Rank test (*n* = 40). **J**-**N** The results of correlation between the expression level of Circ_0001187 and white blood cell, hemoglobin, platelets, BM% and PB% in ND AML patients. Data were analyzed using Pearson correlation. **O** Relative expression of Circ_0001187 in age was measured by qRT-PCR. **P** Diagnostic values of Circ_0001187 in AML patients. AUC: Area under the ROC curve. **Q** The sequence length of spliced mature Circ_0001187 derived from the linear DOPEY2 mRNA is 301 bp. And the results of Sanger sequencing from the head-to-tail splicing in the RT-PCR product of Circ_0001187. **R** Expression levels of Circ_0001187 and linear DOPEY2 mRNA in THP-1 AML cells treated with actinomycin D by qRT-PCR. **S** The qRT-PCR expression results of Circ_0001187 and DOPEY2 using random primer and Oligo dT primer in THP-1 AML cells. **T** Theagarose gel electrophoresis results of Circ_0001187 PCR products of cDNA or gDNA using convergent and divergent primers in THP-1 and Molm-13 AML cells. **U** RNA FISH analysis for Circ_0001187 in THP-1 and Molm-13 AML cells. Scale bars = 10 μm. *p < 0.05; ****p < 0.0001. HCR: HematologicalComplete remission; Ctrl: Control; ND: Newly diagnosed; R/R: relapsed-refractory; ns: Not significant; qRT-PCR: Quantitative reverse transcription PCR

We reanalyzed the differentially expressed circRNAs in AML patients with poor or favorable risk status compared to controls by applying the sequence data of cytogenetically normal AML (CN-AML) patients and healthy controls from our previous circRNA microarray platform study dataset (GEO number: GSE94591) [6]. Based on the fold changes and *P* values of differential expression, the 10 novel circRNAs notated as “AML risk-status related” has been detected, and 3 circRNAs (hsa_circ_0001187, hsa_circ_0011929, and hsa_circ_0000973) satisfied the condition that they had higher expression in cells (Fig. 1A-E, Supplemental Fig. 1A). We further validated their expression in large-scale samples and found that only the expression of Circ_0001187 was significantly decreased in ND AML patients and increased in HCR patients compared with controls, while no significant change was found for the other two circRNAs (Fig. 1F, Supplemental Fig. 1C-D). Moreover, in R/R patients, Circ_0001187 expression was downregulated. To further explore the effect of chemotherapy on Circ_0001187, we followed seven AML patients who received standard chemotherapy until they achieved HCR. We found that the expression level of Circ_0001187 in the HCR stage was significantly increased compared to that in the ND stage (Fig. 1G), indicating that it has potential diagnostic value and antileukemic activity against AML.

Furthermore, we analyzed the correlation between Circ_0001187 expression and the clinical characteristics of patients with AML. Our results showed that Circ_0001187 was downregulated in poor-risk AML patients. Low Circ_0001187 expression exhibited strikingly inferior overall compared to those with high Circ_0001187 expression and 1 patient received transplantation, but this does not affect the overall trend of the survival curve (F ig. 1H-I). Moreover, we analyzed the relationship between Circ_0001187 levels and blood or bone marrow indexes. The results showed that the expression level of Circ_0001187 was negatively correlated with the peripheral white blood cell (WBC) count and the BM or peripheral leukemic blast count but was positively correlated with the hemoglobin level and platelet count (Fig. 1J-N). In addition, we found that its expression was significantly lower in elderly patients, but showed no statistical association with sex (Fig. 1O, Supplemental Fig. 1E). Importantly, the receiver operating characteristic (ROC) curve indicated that it has potential diagnostic value in distinguishing individuals with AML from healthy individuals (Fig. 1P). These results suggest that the decreased expression of Circ_0001187 contributes to poor prognosis and may be a biomarker for the diagnosis and prognosis of AML.

### Characteristics of Circ_0001187 in AML cells

Given the clinical importance of Circ_0001187, we investigated its characteristics in AML. First, we assessed the exon structure of Circ_0001187 and found that it is located at chr21:37619814–37620866 and is derived from exons 20 to 21 of the DOPEY2 gene with a spliced length of 301 bp. We then confirmed the head-to-tail splicing structure of Circ_0001187 by qRT-PCR followed by Sanger sequencing using THP-1 cells (Fig. 1Q). Next, we observed that endogenous Circ_0001187 in THP-1 cells was resistant to digestion by RNase R, which specifically degraded linear RNAs but not circRNAs, further suggesting that Circ_0001187 is a closed-loop structure (Supplemental Fig. 1F). Moreover, owing to its circular structure, we found that Circ_0001187 was more stable than linear DOPEY2 mRNA in AML cells after actinomycin D (a transcription inhibitor) treatment (Fig. 1R, Supplemental Fig. 1G). Circ_0001187 could be amplified by divergent primers in cDNA, but not in genomic DNA, further validating that Circ_0001187 is derived from head-to-tail splicing instead of trans-splicing or genomic rearrangements (Fig. 1T). Due to the lack of a poly (A) tail in circRNAs, Circ_0001187 was nearly undetectable when oligo (dT) primers were used for PCR, whereas its linear DOPEY2 transcript could be identified with both random hexamer and oligo (dT) primers (Fig. 1S, Supplemental Fig. 1H). Furthermore, we detected the subcellular localization of Circ_0001187 in AML cells. Nuclear and cytoplasmic fractionation extracts and FISH assays confirmed that Circ_0001187 was predominantly localized to the cytoplasm of AML cells (Fig. 1U, Supplemental Fig. 1I-K). Taken together, these results demonstrate the presence of Circ_0001187 in AML cells, which is a bonafide circRNA mainly located in the cytoplasm.

### Circ_0001187 suppresses AML progression in vitro and in vivo

**Fig. 2:**
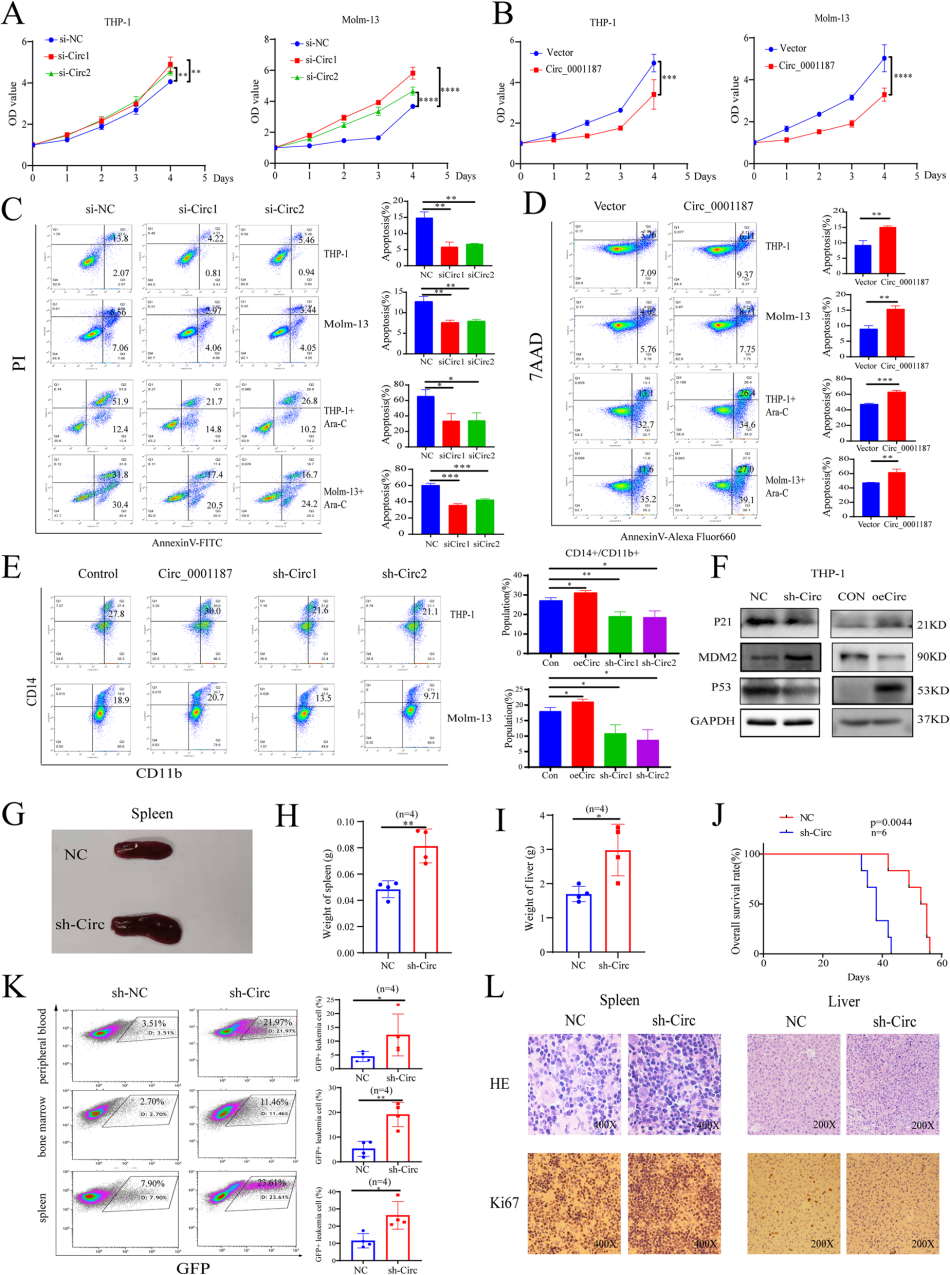
Circ_0001187 suppresses AML progression in vitro and in vivo. **A**-**B** The effect of Circ_0001187 knockdown or overexpression on the proliferation of THP-1 and Molm-13 AML cells by CCK-8 assay. **C**, **D** The effect of Circ_0001187 knockdown or overexpression on the apoptosis of THP-1 and Molm-13 AML cells treated with Ara-c(1uM, 24 h) or not by flow cytometry. **E** THP-1 and Molm-13 AML cells were as sensitive to Circ_0001187 knockdown or overexpression in terms of cell differentiation. **F** Western blot results of P21, MDM2 and P53 in THP-1 cells with knockdown or overexpressed Circ_0001187. **G**-**I** The results of spleen and liver weight of Circ_0001187 knockdown AML mice. **J** Kaplan–Meier analysis of the overall survival of mice treated with sh-Circ or NC. **K** The results of GFP^+^ leukemia cell frequencies in spleen, bone marrow and peripheral blood of mice treated with sh-circRNA or NC. **L** H&E staining showing infiltration of leukemic cells in the spleen and liver of mice engrafted with sh-Circ AML cells compared with that in control mice. And the results of Ki67 staining in the spleen and liver of mice engrafted with sh-Circ AML cells. *p < 0.05; **p < 0.01; ***p < 0.001; ****p < 0.0001

To investigate the biological function of Circ_0001187 in AML, we designed and constructed two siRNAs specifically targeting the backsplice junction region, as well as the full-length vector of Circ_0001187. We identified the expression of Circ_0001187 in seven AML cell lines by qRT-PCR, and finally selected THP-1 and Molm-13 for the following experiments (Supplemental Fig. 1B). The expression analysis using qRT-PCR showed that siRNAs could significantly downregulate Circ_0001187 expression, while the Circ_0001187 vector upregulated its expression in AML cells (Supplemental Fig. 2A-B). We found that downregulation of Circ_0001187 significantly promoted AML cell proliferation, decreased cell apoptosis, and reduced drug sensitivity in AML cells, whereas overexpression of Circ_0001187 inhibited AML proliferation, increased cell apoptosis, and enhanced drug sensitivity in AML cells (Fig. 2A-D, supplemental Fig. 2C). We further assessed the effect of Circ_0001187 overexpression or knockdown on AML cell differentiation. As expected, enforced expression of Circ_0001187 upregulated CD11b and CD14 expression and induced a more mature macrophage-like morphology in AML cells (Fig. 2E). Subsequently, we performed RNA-seq (Supplemental Fig. 2D) to explore the associated mechanisms using Circ_0001187-knockdown AML cells along with controls. After decreasing Circ_0001187 expression, we found that the genes were significantly enriched in many KEGG pathways involved in tumor pathogenesis, including cell proliferation regulation pathways (such as the cell cycle and DNA replication) and pathways associated with apoptosis regulation (p53 signaling pathway) (Supplemental Fig. 2E). Gene set enrichment analysis (GSEA) also showed that cancer-promoting signaling pathways (MYC and p53 signaling pathways) were significantly enriched (Supplemental Fig. 2F-G). Our western blotting results showed that knockdown of Circ_0001187 resulted in a dramatic increase in the expression of oncogenes (MDM2) and sharply decreased the expression of apoptosis-associated genes (p53 and P21) in AML cells, and the opposite trend was observed by Circ_0001187 overexpression in AML cells (Fig. 2F).

To further explore the function of Circ_0001187 in AML in vivo, NOD-Prkdcscid-Il2rgem1IDMO (NPI) mice were intravenously injected with sh-Circ_0001187-GFP AML cells and control AML cells. The AML mice were sacrificed after 40 days, and our results showed that the mRNA level of Circ_0001187 in the BM was significantly decreased in sh-Circ_0001187-GFP AML mice compared with Ctrl-GFP AML mice (Supplemental Fig. 3A). The liver and spleen showed higher weights in sh-Circ_0001187-GFP AML mice (F ig. 2G-I, Supplemental Fig. 3B). Importantly, sh-Circ_0001187 AML mice had a significantly shorter lifespan than Ctrl-GFP AML mice [median 38 (range 33–43) days vs. median 54 (range 42–56) days; *P* = 0.0044] (Fig. 2J). Consistent with these findings, flow cytometric analysis showed that the percentages of GFP^+^ leukemia cells in the peripheral blood, bone marrow, and spleen of sh-Circ_0001187-GFP AML mice were significantly higher than those of Ctrl-GFP AML mice (Fig. 2K). Furthermore, we used hematoxylin and eosin (HE) and Ki67 immunohistochemical staining to evaluate the infiltration of leukemia cells in the spleen and liver. As expected, leukemia cell infiltration was aggravated in sh-Circ_0001187-GFP AML mice compared with that in Ctrl-GFP AML mice (Fig. 2L). Taken together, these results suggest that Circ_0001187 suppresses AML progression, both in vitro and in vivo.

### Decreased Circ_0001187 promoting AML progression by inhibiting ubiquitin-proteasomal degradation of METTL3 and increased m^6^A modification level

**Fig. 3:**
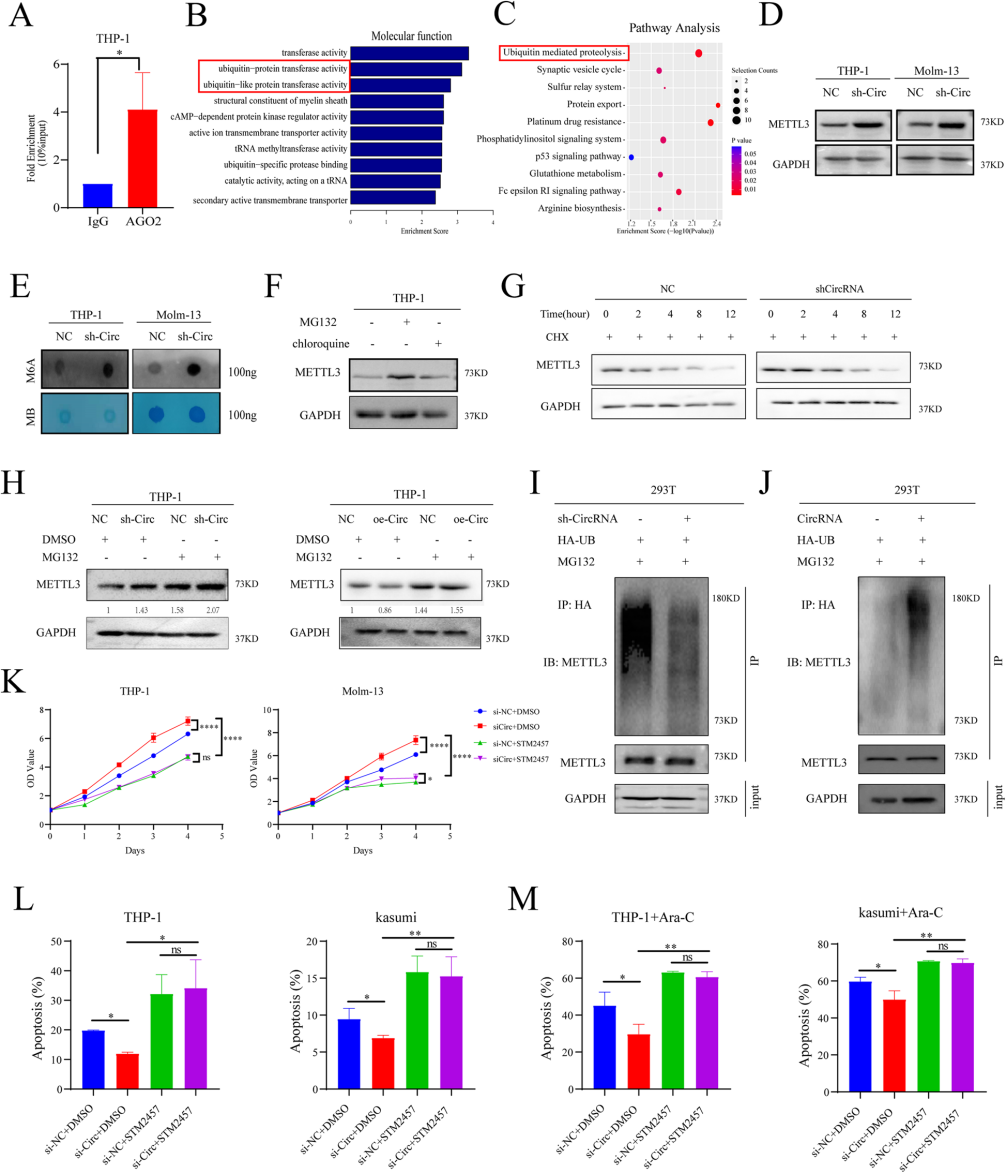
Circ_0001187 facilitates AML suppression by promoting ubiquitin-proteasomal degradation of METTL3 and decreasing m6A modification level. **A** AGO2 RIP assay using AGO2 antibody in THP-1 cells lysates. **B**-**C** The results of the GO enrichment and pathways analysis from RNA pull-down in THP-1 cells. **D** Western blot results of METTL3 in THP-1 and Molm-13 AML cells treated by sh-Circ compared with negative control. **E** The results of m.^6^A dot blot in THP-1 and Molm-13 cells treated by sh-Circ compared with negative control. **F** Western blot results of METTL3 in 293 T cell treated with MG132 (10 μM, 6 h) or chloroquine (100 μM, 6 h). **G** Western blot results of METTL3 in THP-1 cells with Circ_ 0001187 knockdown treated with 20 μg/ml CHX at different times. **H** Western blot results of METTL3 in THP-1 cells with Circ_ 0001187 knockdown or overexpression treated by DMSO or MG132 (10 μM, 10 h). **I**-**J** IP results of METTL3 ubiquitination in 293 T cells. **K**-**M** The effect of Circ_0001187 knockdown or Circ_0001187/ METTL3 inhibitor STM2457 on the proliferation or apoptosis of THP-1 and Molm-13 cells treated with Ara-c (1 μM, 24 h) or not. Data were analyzed using unpaired t-test. *p < 0.05; **p < 0.01; ***p < 0.001; ****p < 0.0001

As Circ_0001187 plays a vital suppressive role in AML progression, we further explored its regulatory mechanism. The AGO2 RIP assay showed that Circ_0001187 could functions via a ceRNA mechanism (Fig. 3A). We then performed a pull-down assay using a biotin-coupled Circ_0001187 probe to investigate sponge-binding miRNAs. We identified 565 differential Circ_0001187-binding miRNAs with a fold change of > 2.0 in the experimental group compared to the negative control group (Supplemental Fig. 3C). Next, we selected six highly expressed miRNAs, identified the top 100 target genes they regulated, and predicted the potential molecular pathways of Circ_0001187 in AML through GO enrichment analysis and KEGG pathway analysis (Supplemental Fig. 3D-F). As KEGG analysis revealed that the target genes of Circ_0001187 were most enriched in the ubiquitin-mediated proteolysis pathway (Fig. 3B-C). We speculate that Circ_0001187 inhibits AML progression via the ubiquitin–proteasome pathway.

Moreover, to identify the potential proteins regulated by Circ_0001187, we lysed proteins from Circ_0001187-downregulated THP-1 cells along with control cells and performed Coomassie blue staining after electrophoresis. We excised the differentially expressed band at approximately 73kD and analyzed it using mass spectrometry (Supplemental Fig. 4A). The results showed that METTL3 protein expression was higher in Circ_0001187-downregulated cells. Furthermore, we performed western blotting and confirmed that the expression level of METTL3 was significantly increased after decreasing Circ_0001187 in AML cells (Fig. 3D). Given that METTL3 is a typical methyltransferase, we explored whether Circ_0001187 could alter the m^6^A methylation level of mRNAs by regulating METTL3 in AML cells. The results of the m^6^A dot blot assay showed that downregulation of Circ_0001187 resulted in a significant increase in mRNA m^6^A modification levels in AML cells (Fig. 3E). It has also been reported that the MYC signaling is a major pathway affected by m6A modification in AML [9, 14], which consistently with our GSEA analysis by using the ranked lists of differentially expressed transcripts in our RNA-seq data upon knockdown Circ_0001187 (Supplemental Fig. 2G). Our analytic results showed that the levels of MYC, MYB and ITGA4, the reported exact mRNA targets of m6A modification in AML [17], are significantly altered by circ_0001187 (Supplemental Fig. 4E).

METTL3 has been reported to promote the initiation and progression of AML by depositing m^6^A modifications on critical transcripts [18]. As expected, our results showed that downregulating Circ_0001187 markedly increased the protein level of METTL3 but not the mRNA level of METTL3, suggesting that posttranscriptional regulation but not transcriptional regulation might be involved in the increase in METTL3 protein levels (Fig. 3D, Supplemental Fig. 4C). And we confirmed that METTL3 protein was mainly degraded via the proteasome pathway but not the lysosome pathway (Fig. 3F). We next examined whether METTL3 ubiquitin/proteasome-dependent degradation is regulated by the expression of Circ_0001187 (Fig. 3G, Supplemental Fig. 4D). Our results showed that the increased METTL3 expression caused by Circ_0001187 knockdown could be restored by the proteasome inhibitor MG132. And the opposite trend was observed by Circ_0001187 overexpression in AML cells (Fig. 3H). Furthermore, we knocked down Circ_0001187 and found that METTL3 ubiquitination was dramatically decreased compared with the negative control group (Fig. 3I). Moreover, Circ_0001187 overexpression significantly increased the ubiquitination level of METTL3 in AML cells (Fig. 3J). These results demonstrate that Circ_0001187 enhances METTL3 protein degradation via the ubiquitin/proteasome-dependent degradation pathway, which further decreases the mRNA m^6^A modification level in AML cells.

Subsequently, to validate that METTL3 is truly a functional target of Circ_0001187, we investigated whether METTL3 could mediate the biological effects of Circ_0001187 on AML progression. Results showed that the increase in proliferation, decrease in the apoptosis rate and reduction in the sensitivity to drugs caused by downregulated Circ_0001187 were reversed by METTL3 inhibitor STM2457 (Fig. 3K-M) [19]. These results demonstrate that Circ_0001187 inhibits AML progression by regulating METTL3.

### Circ_0001187-induced METTL3 degradation is mediated by E3 ubiquitin ligase RNF113A

**Fig. 4:**
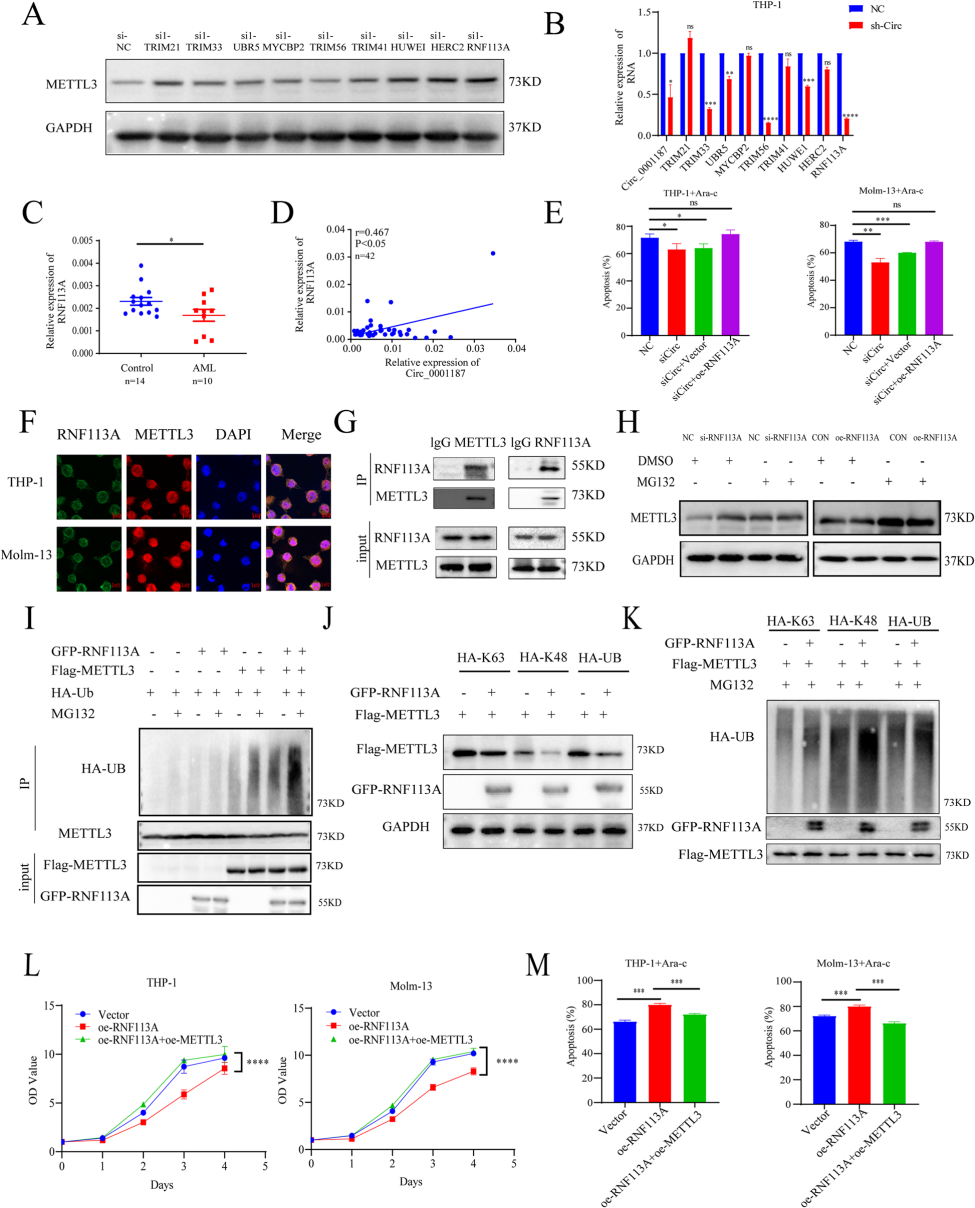
RNF113A functions as an E3 ubiquitin ligase to mediate Circ_0001187-induced METTL3 degradation. **A** Western blot results of METTL3 in THP-1 cells transduced with the siRNA of potential E3 ligases respectively compared with negative control. **B** The expression levels of 9 potential E3 ligases in THP-1 cells after downregulating Circ_0001187 with siRNA through qRT-PCR. **C** Expression levels of RNF113A in AML patients and healthy controls through qRT-PCR. **D** The results of correlation between the expression level of RNF113A and Circ_0001187. **E** The effect of si-Circ or si-Circ/oe-RNF113A on the apoptosis of THP-1 and Molm-13 cells cultured with Ara-C (1 μM, 24 h). **F** IF assays showing the co-localization of RNF113A with METTL3 in THP-1 and Molm-13 AML cells. Scale bar, 20 μm. **G** Co-IP showed the binding of METTL3 with RNF113A in 293 T cells. **H** Western blot results of METTL3 in 293 T cells with the RNF113A knockdown or overexpression treated with DMSO or MG132 (10 μM, 6 h). **I** The results of METTL3 ubiquitination in 293 T cells expressed GFP-RNF113A, Flag-METTL3 and HA-ubiquitin and treatment with or without MG132 (10 μM, 6 h). **J** Western blot results of investigate the form of polyubiquitin chains linked to METTL3. **K** The results of RNF113A-induced METTL3 ubiquitination detected in WT HA-UB and K48 transfected cells. **L** The effect of RNF113A overexpression or RNF113A/METTL3 overexpression on the proliferation of THP-1 and Molm-13 cells. **M** The effect of RNF113A overexpression or RNF113A/METTL3 overexpression on the apoptosis of THP-1 and Molm-13 cells cultured with Ara-C (1 μM, 24 h). *p < 0.05; **p < 0.01; ***p < 0.001; ****p < 0.0001; ns: Not significant

Although accumulating evidence has demonstrated that METTL3 plays a critical role in promoting AML progression as an m^6^A methyltransferase [14, 19], it has not been reported whether E3 ubiquitin ligase can mediate the ubiquitination of METTL3 in AML. To identify METTL3-related E3 ligases, we first expressed Flag-METTL3 in AML cells and performed mass spectrometric analysis of METTL3-immunoprecipitated complexes in AML cells. Nine potential E3 ligases were identified by mass spectrometric analysis. We then downregulated these nine E3 ligases by employing siRNA and observed a substantial increase in the protein level of METTL3 after inhibiting the expression of the E3 ligases RNF113A, TRIM21, HUWE1, and HERC2 with siRNA transfection (Fig. 4A, Supplemental Fig. 4G). Moreover, our results showed that Circ_0001187 downregulation significantly decreased the expression of RNF113A and HUWE1, especially RNF113A (Fig. 4B). Indicating that the E3 ligases RNF113A might be involved in Circ_0001187-induced METTL3 protein degradation.

To explore the clinical relevance of RNF113A, we determined its expression in the BM of AML patients and found that the level of RNF113A decreased in AML patients (Fig. 4C). Consistently, an overall marginal decrease of RNF113A expression in AML was also observed in The Cancer Genome Atlas (TCGA) database (Supplemental Fig. 4H). To assess RNF113A function in leukemia, we first knocked down RNF113A in myeloid leukemia cells using two siRNAs. RNF113A knockdown significantly promoted cellular growth and inhibited apoptosis of leukemia cells (Supplemental Fig. 4I-J). As RNF113A expression positively correlated with Circ_0001187 (Fig. 4D), we propose that RNF113A is a potential downstream target of Circ_0001187. Thus, we over-expressed RNF113A in Circ_0001187-knockdown leukemia cells and found that RNF113A restoration partially rescued the drug sensitivity defects caused by Circ_0001187 deficiency (Fig. 4E).

RNF113A contains a RING domain, which has been shown to functions as an E3 ubiquitin ligase [20, 21]. As expected, immunofluorescence analysis showed that METTL3 colocalized with RNF113A in the cytoplasm of AML cells (Fig. 4F). The results of reciprocal co-immunoprecipitation assays confirmed a physical interaction between METTL3 and RNF113A in the cells (Fig. 4G). Moreover, our results showed that RNF113A overexpression decreased the levels of METTL3 protein, while knockdown of RNF113A with siRNA increased the level of METTL3, and these effects could be reversed by MG132 treatment (Fig. 4H). Moreover, the results of the IP assay showed that the ubiquitination level of METTL3 dramatically increased after overexpression of RNF113A (Fig. 4I). These results suggest that RNF113A regulates METTL3 expression via ubiquitination/proteasome degradation. Next, to investigate the form of polyubiquitin chains linked to METTL3, we performed IP followed by western blotting using cells transfected with WT HA-ubiquitin or its mutants, K48 and K63, which have only one lysine residue in ubiquitin at positions 48 and 63, respectively. The results showed that transfection of WT HA-UB and its mutant K48 could promote the degradation of METTL3, whereas HA-K63 could not, and these effects could be promoted by the overexpression of RNF113A (Fig. 4J). Accordingly, RNF113A-induced METTL3 ubiquitination could be easily detected in WT HA-UB and K48 transfected cells, while there was much less METTL3 ubiquitination in K63 transfected cells, suggesting that RNF113A mainly mediates K48-linked METTL3 ubiquitination (Fig. 4K). For the functional study, we found that overexpression of RNF113A significantly inhibited AML cell proliferation, enhanced drug sensitivity, and effectively alleviated the leukemia-promoting effects caused by METTL3 overexpression *in vitro* (Fig. 4L-M). Taken together, our results suggest that Circ_0001187 facilitates RNF113A-mediated ubiquitination and degradation of METTL3.

### Circ_0001187 acts as a sponge to enhance RNF113A expression via Circ_0001187/ miR-499a-5p/ RNF113A axis

As our results showed that Circ_0001187 regulates RNF113A through the ceRNA pathway, we further investigated the key miRNAs underlying this molecular mechanism. We combined these analytical results with the experimental results of pull-down assays with Circ_0001187 and found that miR-499a-5p may be the mediator between Circ_0001187 and RNF113A (Supplemental Fig. 3D).

**Fig. 5:**
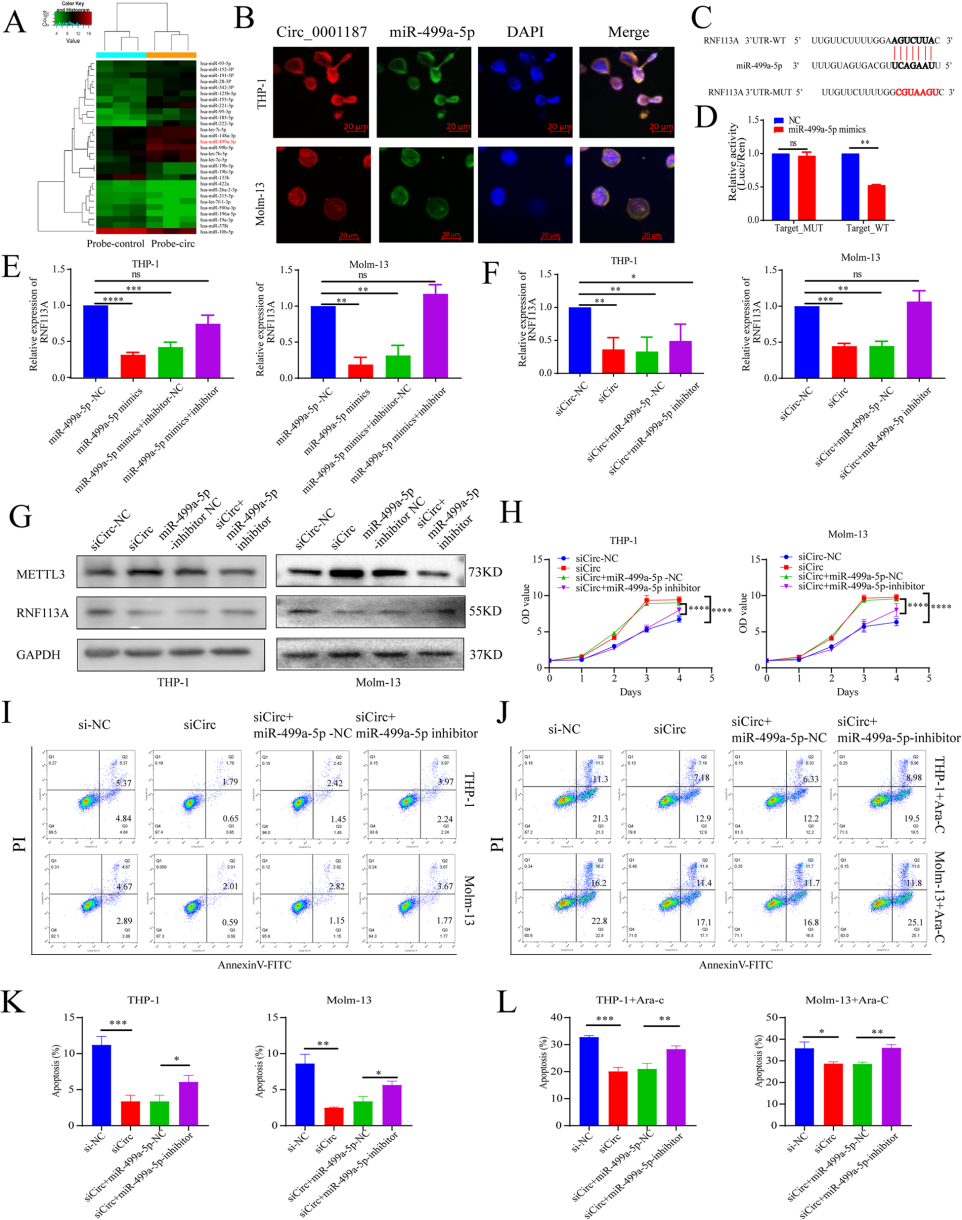
Circ_0001187 acts as a sponge to enhance RNF113A expression via Circ_0001187/ miR-499a-5p/ RNF113A axis. **A** Heatmap for differentially expressed miRNAs via RNA pulldown assay by probe-Circ_0001187. **B** RNA FISH showing colocalization of Circ_0001187 and miR-499a-5p in THP-1 and Molm-13 cells. Scale bars, 20um. **C** RNF113A contains conserved target sites of miR-499a-5p by using the Target Scan and its mutations. **D** The results of dual-luciferase reporter assay for miR-499a-5p and RNF113A. **E** The expression levels of RNF113A in THP-1 and Molm-13 cells transfected with miR-499a-5p mimics or mimics/inhibitor respectively through qRT-PCR. **F** The expression levels of RNF113A in THP-1 and Molm-13 cells transfected with si-Circ or si-Circ/miR-499a-5p inhibitor through qRT-PCR. **G** Western blot results of RNF113A and METTL3 in THP-1 and Molm-13 cells transfected with si-Circ or si-Circ/miR-499a-5p inhibitor. **H** The effect of si-Circ or si-Circ/miR-499a-5p inhibitor on the proliferation of THP-1 and Molm-13 cells. **I**, **K** The effect of si-Circ or si-Circ/miR-499a-5p inhibitor on the apoptosis of THP-1 and Molm-13 cells. **J**, **L** The effect of si-Circ or si-Circ/miR-499a-5p inhibitor on the apoptosis of THP-1 and Molm-13 cells cultured with Ara-C (1 μM, 24 h). *p < 0.05; **p < 0.01; ***p < 0.001; ****p < 0.0001; ns: Not significant

MiR-499a-5p has been reported to be involved in many human cancers [22, 23], and it was found to be upregulated in AML samples [24]. Differential miRNA clustering results from miRNA-pulldown sequencing showed that miR-499a-5p was significantly enriched with Circ_0001187 (Fig. 5A). FISH assay showed that Circ _0001187 and miR-499a-5p were co-localized in the cytoplasm of AML cells (Fig. 5B, Supplemental Fig. 5A). Next, to confirm the binding of miR-499a-5p to RNF113A, we performed a dual-luciferase reporter assay and further validated that RNF113A is the direct target gene of miR-499a-5p (Fig. 5C-D). We found that the mRNA level of RNF113A was substantially decreased in AML cells after transfection with miR-499a-5p mimics, whereas it was notably enhanced after transfection with the inhibitor (Fig. 5E). Moreover, knockdown of Circ_0001187 with siRNA decreased RNA113A expression at the mRNA and protein levels, and these effects were reversed by the miR-499a-5p inhibitor (Fig. 5F-G). In addition, our results also showed that protein levels of METTL3 were significantly upregulated by Circ_0001187 knockdown but notably decreased after transfecting cells with the miR-499a-5p inhibitor (Fig. 5G). These data demonstrate the ceRNA mechanism of the Circ_0001187/miR-499a-5p/RNF113A/METTL3 pathway in AML cells.

Next, we explored the function of this pathway in AML cells. We first treated AML cells with miR-499a-5p mimics and found that AML cell proliferation was increased, while apoptosis was inhibited (Supplemental Fig. 5B-C). Furthermore, to explore the biological effects of Circ_0001187 on miR-499a-5p, we performed rescue experiments in which the miR-499a-5p inhibitor was used after transfecting si-Circ _0001187 into AML cells. Our results showed that the miR-499a-5p inhibitor significantly attenuated apoptosis or sensitivity to drugs and enhanced the proliferation of AML cells by knocking down Circ_0001187 (Fig. 5H-L). In summary, these findings indicate that Circ_0001187 exerts an anti-leukemic effect via the miR-499a-5p/RNF113A pathway.

### Circ_0001187 is epigenetically regulated by promoter DNA methylation and histone deacetylation

**Fig. 6:**
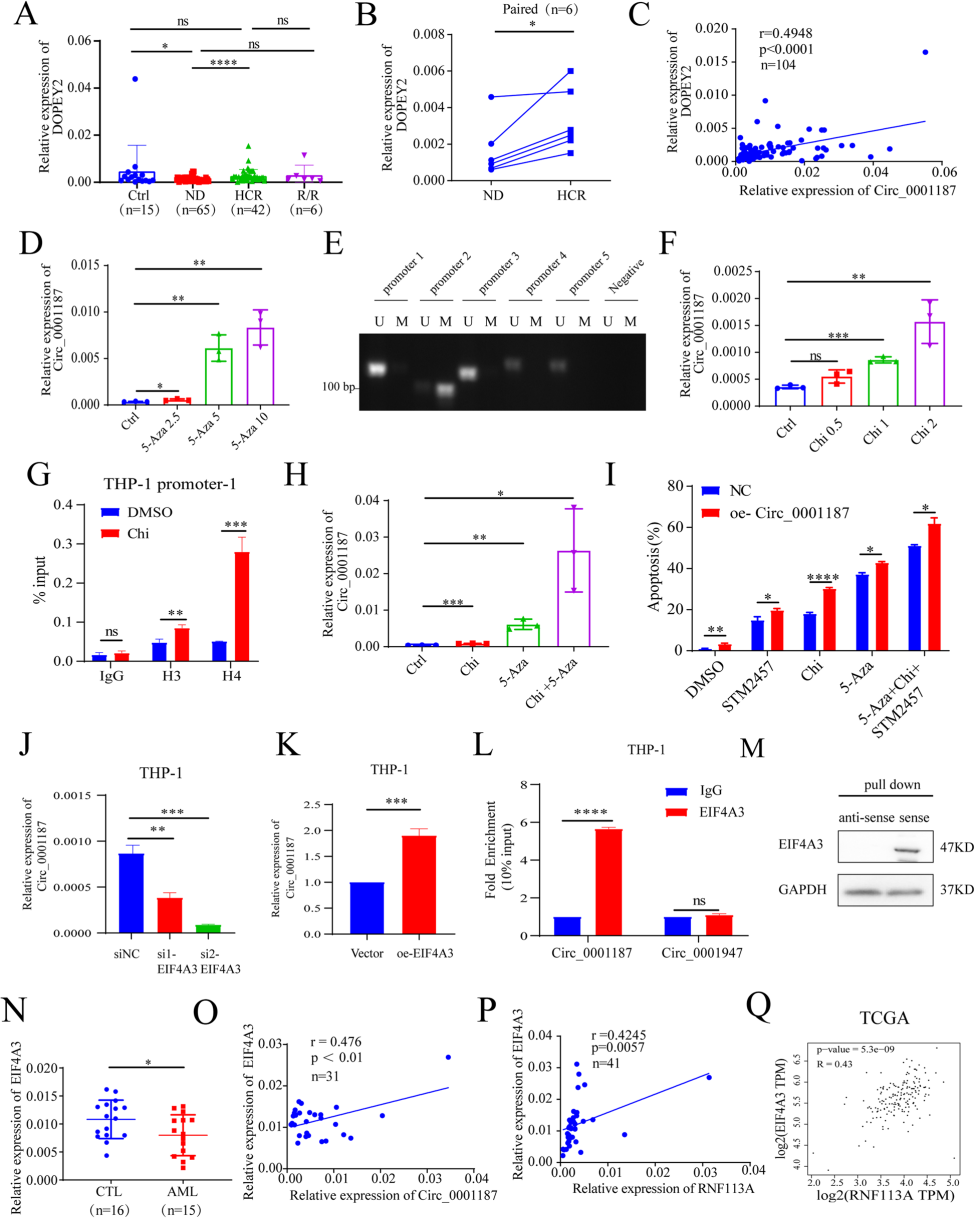
Circ_0001187 is epigenetically regulated, and combining chidamide and 5-azacytidine with METTL3 inhibitor synergistically suppresses AML cells. **A** The expression results of DOPEY2 in AML patients and healthy controls through qRT-PCR. **B** The expression results of DOPEY2 in paired samples from six follow-up AML patients at ND and HCR stage. **C** The results of correlation between the expression levels of Circ_0001187 and DOPEY2. **D** The expression levels of Circ_0001187 in AML cells cultured with 5-azacytidine (2.5, 5 and 10 μM, 24 h) or not by qRT-PCR. **E** The results of MSP by Agarose gel electrophoresis. **F** The expression levels of Circ_0001187 in AML cells cultured with chidamide (0.5, 1 and 2 μM, 24 h) or not by qRT-PCR. **G** ChIP‒qPCR showing the effect of chidamide on the histone acetylation levels of Circ_0001187 by promoter-1. **H** The expression levels of Circ_0001187 in AML cells cultured with CHi or 5-Aza or combination by qRT-PCR. **I** The effect of oe-Circ_0001187 on the apoptosis of AML cells cultured with METTL3 inhibitor, CHi or 5-Araz or combination. **J**-**K** The expression levels of Circ_0001187 in THP-1 cells transfected with si1/si2-EIF4A3 or oe-EIF4A3. **L** RIP assay to verify the physical interaction between EIF4A3 and the pre-mRNA of Circ_0001187 and Circ_0001947. **M** The results of RNA pull-down assays. **N** The expression level of EIF4A3 in AML patients and healthy controls through qRT-PCR. **O** The results of correlation between the expression levels of Circ_0001187 and EIF4A3. **P**-**Q** The results of correlation between the expression levels of RNF113A and EIF4A3. HCR: Hematological Complete remission; Ctrl: Control; ND: Newly diagnosed; R/R: relapsed-refractory; *p < 0.05; **p < 0.01; ***p < 0.001; ****p < 0.0001; ns: Not significant

The important role of Circ_0001187 compelled us to investigate its silencing mechanisms in AML, which could help develop effective Circ_0001187-activating agents. The biogenesis of circRNAs has been reported to be regulated both co and post-transcriptionally in cells [5, 7]. Our results showed that Circ_0001187 and its linear DOPEY2 mRNA showed a similar decrease in expression in ND and R/R AML patients (Fig. 6A-B), and were positively correlated with each other (Fig. 6C). Therefore, we speculated that downregulation of Circ _0001187 may be regulated co-transcriptionally in AML.

Therefore, we conducted an in-depth study of promoter DNA methylation and histone acetylation to explore the suppression regulatory mechanisms of Circ_0001187 in AML. We confirmed that there were potential CpG islands in the promoter region of Circ_0001187 using CpG island Searcher (http://cpgislands.usc.edu/). Furthermore, we treated AML cells with 5-azacytidine (methyltransferase inhibitor), and the results showed that this demethylated drug significantly enhanced Circ_0001187 expression (Fig. 6D). We further used MSP to confirm that the Circ_0001187 promoter is highly methylated in AML cells (Fig. 6E).

To address the role of histone modifications in Circ_0001187 silencing in AML, we treated AML cells with chidamide (HDAC 1,2,3 inhibitor) and found that the expression of Circ_0001187 was significantly increased following chidamide treatment (Fig. 6F). We then assessed the effect of chidamide on the histone acetylation levels of Circ_0001187 using ChIP‒qPCR and observed that the levels of histone H3 and H4 acetylation at the Circ_0001187 promoter was significantly increased after chidamide treatment (Fig. 6G, Supplemental Fig. 5D-I). Furthermore, we combined 5-azacytidine with chidamide, and the results showed that they could lead to a much higher expression of Circ_0001187 (Fig. 6H). As METTL3 is the executor of Circ_0001187, we further evaluated whether the combination of METTL3 inhibitor with chidamide or 5-azacytidine could exhibit potent antileukemic activity against AML. As expected, the combination of METTL3 inhibitor with chidamide or 5-azacytidine markedly increased the apoptosis of AML cells (Fig. 6I). In addition, Circ_0001187 overexpression in AML cells significantly promoted apoptosis caused by chidamide or 5-azacytidine treatment, demonstrating the contribution of Circ_0001187 to these antileukemic activities (Fig. 6I). These data suggest that Circ_0001187 can be silenced in AML by both promoter DNA methylation and histone deacetylation.

### Circ_0001187 is post-transcriptionally regulated by RNA-binding protein EIF4A3

As the decrease in Circ_0001187 expression in ND AML patients compared to controls (Ctrl vs. ND, *p* < 0.0001) was more obvious than that in DOPEY2 expression (Ctrl vs. ND, *p* < 0.05) (Figs. 1F and 6A), we speculate that the downregulation of Circ_0001187 may also be regulated by a post-transcriptional mechanism in AML. Therefore, we first explored biogenesis mediated by RNA-binding proteins (RBPs) post-transcriptionally to facilitate the biogenesis of exon-derived circRNAs. We used the CircInteractome (https://circinteractome.nia.nih.gov/) and RBPsuite (http://www.csbio.sjtu.edu.cn/bioinf/RBPsuite/) database to predict the RBPs of Circ_0001187 and found that there simultaneously exists RNA binding protein EIF4A3 (eukaryotic translation initiation factor 4A3) and FMRP (fragile X mental retardation protein). However, only EIF4A3 has multiple binding sites in RBPsuite database (Supplementary Fig. 5J-K). EIF4A3 has been proven to be an RNA helicase and core component of the exon junction complex, which was reported to promote the expression of circRNAs [25, 26]. To clarify the role of EIF4A3 and its relationship with Circ_0001187 in AML, we performed a functional study and found that the expression of Circ_0001187 was significantly decreased when knocked down EIF4A3 in AML cells (Fig. 6J). As expected, overexpression of EIF4A3 led to a remarkable increase in Circ_0001187 expression (Fig. 6K). Next, we performed an RNA immunoprecipitation assay to verify the physical interaction and confirmed that EIF4A3 indeed bound to the pre-mRNA of Circ_0001187, but not to other circular RNA such as Circ_0001947 (Fig. 6L). And the RNA pull-down assay further warrants the interaction of EIF4A3 with circ_0001187 (Fig. 6M). To clarify the clinical relevance of EIF4A3, we further determined its expression and found that EIF4A3 expression was decreased in AML patients compared to that in controls (Fig. 6N). An overall decreasing trend in AML was also observed in TCGA database (Supplemental Fig. 5L). Furthermore, we found that the expression of EIF4A3 was positively correlated with Circ_0001187 or RNF113A in AML patients (Fig. 6O-P). The expression of EIF4A3 was also positively correlated with RNF113A using TCGA database (Fig. 6Q). We also examined the biological effects of EIF4A3 on AML cells. The results showed that overexpression of EIF4A3 inhibited AML cell proliferation and promoted apoptosis. More importantly, all these biological effects induced by overexpression of EIF4A3 were reversed after downregulation of Circ_0001187 (Supplemental Fig. 5M-N). These results demonstrate that Circ_0001187 biogenesis could be regulated by EIF4A3 and that the low level of Circ_0001187 in AML results from a decrease in EIF4A3 expression.

## Discussion

Aberrant expression of circRNAs plays a crucial role in the development of many cancers [20, 21, 27–31]. CircRNAs have recently been reported to promote AML initiation and maintenance [5, 32]. However, the regulatory mechanisms involved in AML remain unclear. Here, we identified a new circRNA, Circ_0001187, which is downregulated in AML patients and significantly associated with a favorable prognosis. We further explored the epigenetic regulatory mechanisms underlying Circ_0001187 silencing by promoter hypermethylation or histone deacetylation and post-transcriptional biogenesis mediated by EIF4A3. Circ_0001187 inhibits AML progression in vitro and in vivo. Importantly, Circ_0001187 facilitated METTL3 ubiquitination and degradation by reducing the repression of miR-499a-5p and further promoting RNF113A expression. We first reported that Circ_0001187 inhibits AML progression by regulating the level of m^6^A modification.

Increasing evidence shows that circRNA expression is associated with clinicopathological features in patients with tumors [26, 27, 31, 33, 34]. In our study, we found that Circ_0001187 was significantly decreased in ND AML patients and increased in patients with HCR. Decreased Circ_0001187 in AML was significantly correlated with poor-risk AML patients, exhibited strikingly shorter overall survival, and had potential diagnostic value. In addition to apoptosis and proliferation, we also observed selective regulation of myeloid differentiation by Circ_0001187 in leukemia cells. And the reason for the significant differences between si-NC and vector groups may be due to that we performed the experiments in different panel and at different time, which caused the obvious differences in OD values, apoptosis rates, or band intensity (P21, MDM2, P53). Our previous studies showed that AML often exhibits a reduction in global circRNA abundance [35, 36]. We investigated the mechanism underlying the downregulation of Circ_0001187 at both the epigenetic and post-transcriptional levels in AML. Epigenetic mechanisms, especially promoter hypermethylation or histone deacetylation, are known to play a key role in the silencing of tumor suppressor genes [37]. In our study, after treatment with HDAC inhibitors or DNA methyltransferase inhibitors, we found that both were efficacious activators of Circ_0001187. In addition, we explored the mechanism underlying the downregulation of Circ_0001187 at the post-transcriptional level in AML. EIF4A3 has recently been reported to be a novel regulator of circRNA biogenesis and plays key roles in various diseases [21, 26, 28, 38]. Our study is the first to show that EIF4A3 is downregulated in AML, and that overexpression of EIF4A3 inhibits proliferation and promotes apoptosis of cells. Our study suggests that promoter hypermethylation, histone deacetylation, and a decrease in EIF4A3 expression contribute to the downregulation of Circ_0001187.

The m^6^A writer METTL3, a potential therapeutic target for AML treatment, has recently been reported to promote AML initiation and maintenance [14]. However, the regulatory mechanisms involved in AML remain unclear. In this study, we identified the regulatory mechanism of Circ_0001187 on METTL3 in AML and found that Circ_0001187 promotes RNF113A-mediated METTL3 degradation and the reduction of m^6^A modification levels. Thus, the decrease in m^6^A modification levels caused by the vigorous activation of Circ_0001187 may account for the specific induction of apoptosis in AML cells. Furthermore, we elucidated the regulatory mechanism of METTL3 by demonstrating that RNF113A is an E3 ligase that mediates METTL3 ubiquitination degradation. Our study is the first to show that the E3 ligase RNF113A is downregulated in AML, and that overexpression of RNF113A inhibits proliferation and promotes apoptosis of cells. Recent studies have shown that proteolysis-targeting chimeras (PROTACs) are a new frontier for therapeutics [39–41], and our data provide a strategy for the RNF113A-targeted degradation of METTL3 in AML.

CircRNAs play functional roles in a variety of biological processes, mostly by acting as miRNA sponges to relieve repression of target mRNAs [30–33, 41, 42]. CircTP63 functions as a ceRNA to promote lung squamous cell carcinoma progression by upregulating FOXM1 [20]. Here, based on both bioinformatic predictions and miRNA-seq data, we proved that Circ_0001187 inhibits AML progression by sponging miR-499a-5p. MiR-499a-5p has been reported to play an important role in several diseases [22, 23, 43, 44]. However, the role of miR-499a-5p in AML remains to be elucidated. Our results suggest that miR-499a-5p inhibits proliferation and promotes apoptosis in AML cells. The abundance of miR-499a-5p was lower in AML cells, and miR-499a-5p was sponged by Circ_0001187, resulting in less miR-499a-5p targeting RNF113A. Identification of the Circ_0001187-miR-499a-5p-RNF113A axis expands our understanding of the underlying mechanism of AML progression.

**Fig. 7:**
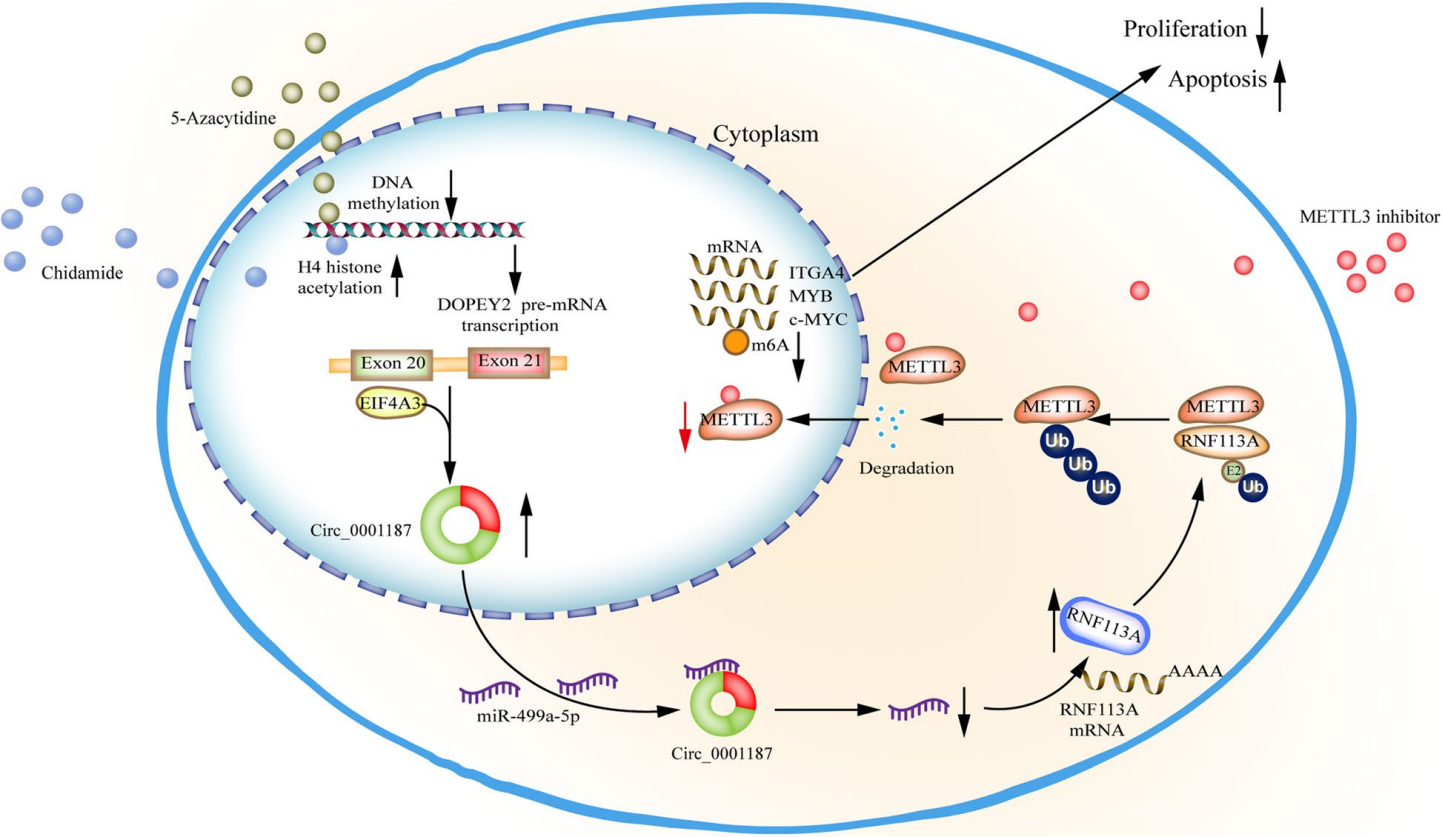
Proposed working model of EIF4A3-induced Circ_0001187 facilitates AML suppression through promoting ubiquitin-proteasomal degradation of METTL3 and decreasing m^6^A modification level mediated by miR-499a-5p/RNF113A pathway

In conclusion, Circ_0001187 is an independent prognostic predictor that contributes to favorable outcomes in AML. Circ_0001187 suppresses AML progression through the miR-499a-5p/RNF113A/METTL3 cascade and subsequent activation of downstream signaling pathways. The tumor-suppressive function of Circ_0001187 suggests that it may have therapeutic value for AML treatment (Fig. 7: Visual Figure).

## Supplementary Information

**Additional file 1: Supplementary Figure 1. **Expression levelsof different circRNA in patients.Expression levels of Circ_0001187 in AML cell lines. The expressions of Circ_0011929 and Circ_0000973 in AML patients and healthy controls.Relative expression of Circ_0001187 in gender was measured by qRT-PCR.Expression levels of Circ_0001187 and linear DOPEY2 mRNA in THP-1 cellsafter being treated with RNase R by qRT-PCR. Data were analyzed using Unpairedt-test.Expression levels of Circ_0001187 and linearDOPEY2 mRNA in Molm-13 AML cells treated with actinomycin D by qRT-PCR. The qRT-PCR expression results of Circ_0001187 and DOPEY2 using randomprimer and Oligo dT primer in Molm-13 AML cells. The qRT-PCR analysis of nuclear and cytoplasmic fractionation extractsin THP-1 andMolm-13 AML cells. The results of RNA FISH by using sense probein THP-1 and Molm-13 AML cells. **p < 0.01; ****p < 0.0001;ns: Not significant. **Supplementary Figure 2.** The qRT-PCR results of Circ_0001187 in THP-1 and Molm-13 cells withCirc_0001187 knockdown or overexpression. The proliferation results of THP-1 cellstransfected si-Circ1 or si-Circ 2 by EDU staining. The expression levelof differential genes by RNA-seq analysis.KEGG pathways in Circ_0001187 knockdown compared to the control group.GSEA analysis for Circ_0001187 knockdown compared to the controlgroup. *p < 0.05; **p < 0.01; ***p < 0.001; ****p < 0.0001. **Supplementary Figure 3.** The qRT-PCR results of Circ_0001187 in mice treatedwith sh-Circ compared with negative control. The spleen weight of AML mice injectedwith THP-1 cells transfected with sh-Circ_0001187-GFP or Ctrl-GFP.Differential expression of miRNA from RNA pull-down.miRNA and target mRNA predictionnetwork of Circ_0001187 via RNA pulldown assay. The results of GO enrichment correspond tomiRNA/mRNA. *p < 0.05; ***p < 0.001. **Supplementary Figure 4.** The results of coomassie blue staining inTHP-1with Circ_0001187 knockdown. The protein METTL3 identified by mass spectrometryanalysis. The qRT-PCR results of METTL3 in THP-1 and Molm-13 AML cells with Circ_0001187 knockdown.Western blot results of METTL3 in 293-T cells treated with 20 μg/ml CHX at different times. The mRNA leve of MYB, MYC and ITGA4 in THP-1and Molm-13AML cells with Circ_0001187 knockdown. The potential METTL3 E3 ligases identifiedby affinity MS.Western blot results of METTL3in THP-1 cells transduced with the siRNA of potentialE3 ligases respectively compared with negative control. The expression levels of RNF113A from TCGA database. The proliferation results of THP-1 and Molm-13cells with RNF113A knockdown by CCK-8 assays. The effect of RNF113A knockdown on theapoptosis of THP-1 and Molm-13 cells by flow cytometry. *p < 0.05; **p< 0.01; ***p < 0.001; ****p < 0.0001; ns: Not significant. **Supplementary Figure 5.** The results of RNA FISH by using sense probe in THP-1 and Molm-13 AML cells. The effect of miR-499a-5p mimics on theproliferation of THP-1 and Molm-13 cells. The effect of miR-499a-5p mimics onthe apoptosis of THP-1 and Molm-13 cells.ChIP‒qPCR showingthe effect of chidamide on the histoneacetylation levels of Circ_0001187 by promoters in THP-1 and Molm-13 cells. TheRNA-bing protein sites matching flanking regions of circRNA from Circintercomedatabase. The results from RBPsuit database. The expression levels of EIF4A3 from TCGAdatabase. The effect of oe-EIF4A3or oe-EIF4A3/siCirc on the proliferation of THP-1 cells. The effect of oe-EIF4A3 or oe-EIF4A3/siCircon the apoptosis of THP-1 cells. *p < 0.05; **p <0.01; ***p < 0.001; ****p< 0.0001; ns: Not significant. **Supplementary Table 1.** Risk classification standard. **Supplementary Table 2.** Primers used for quantitative reversetranscription PCR. **Supplementary Table 3.** The sequences for oligonucleotide transfection. **Supplementary Table 4.** Primer sets for MSP and CHIP

## Data Availability

The datasets used and/or analyzed during the current study are available from the corresponding author upon reasonable request.
