# Supplementary material for: EIF4A3-induced Circ_0001187 facilitates AML suppression through promoting ubiquitin-proteasomal degradation of METTL3 and decreasing m6A modification level mediated by miR-499a-5p/RNF113A pathway
Source: Biomark Res. 2023 Jun 6;11:59. doi: 10.1186/s40364-023-00495-4 (PMC10243067; doi:10.1186/s40364-023-00495-4)
Supplement: Supplementary file 1 — Additional file 1: Supplementary Figure 1. Expression levelsof different circRNA in patients.Expression levels of Circ_0001187 in AML cell lines. The expressions of Circ_0011929 and Circ_0000973 in AML patients and healthy controls.Relative expression of Circ_0001187 in gender was measured by qRT-PCR.Expression levels of Circ_0001187 and linear DOPEY2 mRNA in THP-1 cellsafter being treated with RNase R by qRT-PCR. Data were analyzed using Unpairedt-test.Expression levels of Circ_0001187 and linearDOPEY2 mRNA in Molm-13 AML cells treated with actinomycin D by qRT-PCR. The qRT-PCR expression results of Circ_0001187 and DOPEY2 using randomprimer and Oligo dT primer in Molm-13 AML cells. The qRT-PCR analysis of nuclear and cytoplasmic fractionation extractsin THP-1 andMolm-13 AML cells. The results of RNA FISH by using sense probein THP-1 and Molm-13 AML cells. **p < 0.01; ****p < 0.0001;ns: Not significant. Supplementary Figure 2. The qRT-PCR results of Circ_0001187 in THP-1 and Molm-13 cells withCirc_0001187 knockdown or overexpression. The proliferation results of THP-1 cellstransfected si-Circ1 or si-Circ 2 by EDU staining. The expression levelof differential genes by RNA-seq analysis.KEGG pathways in Circ_0001187 knockdown compared to the control group.GSEA analysis for Circ_0001187 knockdown compared to the controlgroup. *p < 0.05; **p < 0.01; ***p < 0.001; ****p < 0.0001. Supplementary Figure 3. The qRT-PCR results of Circ_0001187 in mice treatedwith sh-Circ compared with negative control. The spleen weight of AML mice injectedwith THP-1 cells transfected with sh-Circ_0001187-GFP or Ctrl-GFP.Differential expression of miRNA from RNA pull-down.miRNA and target mRNA predictionnetwork of Circ_0001187 via RNA pulldown assay. The results of GO enrichment correspond tomiRNA/mRNA. *p < 0.05; ***p < 0.001. Supplementary Figure 4. The results of coomassie blue staining inTHP-1with Circ_0001187 knockdown. The protein METTL3 identified by mass spectrometryanalysis. The qRT- [file 40364_2023_495_MOESM1_ESM.docx]

**Supplementary**

**Supplementary Figure 1**

**
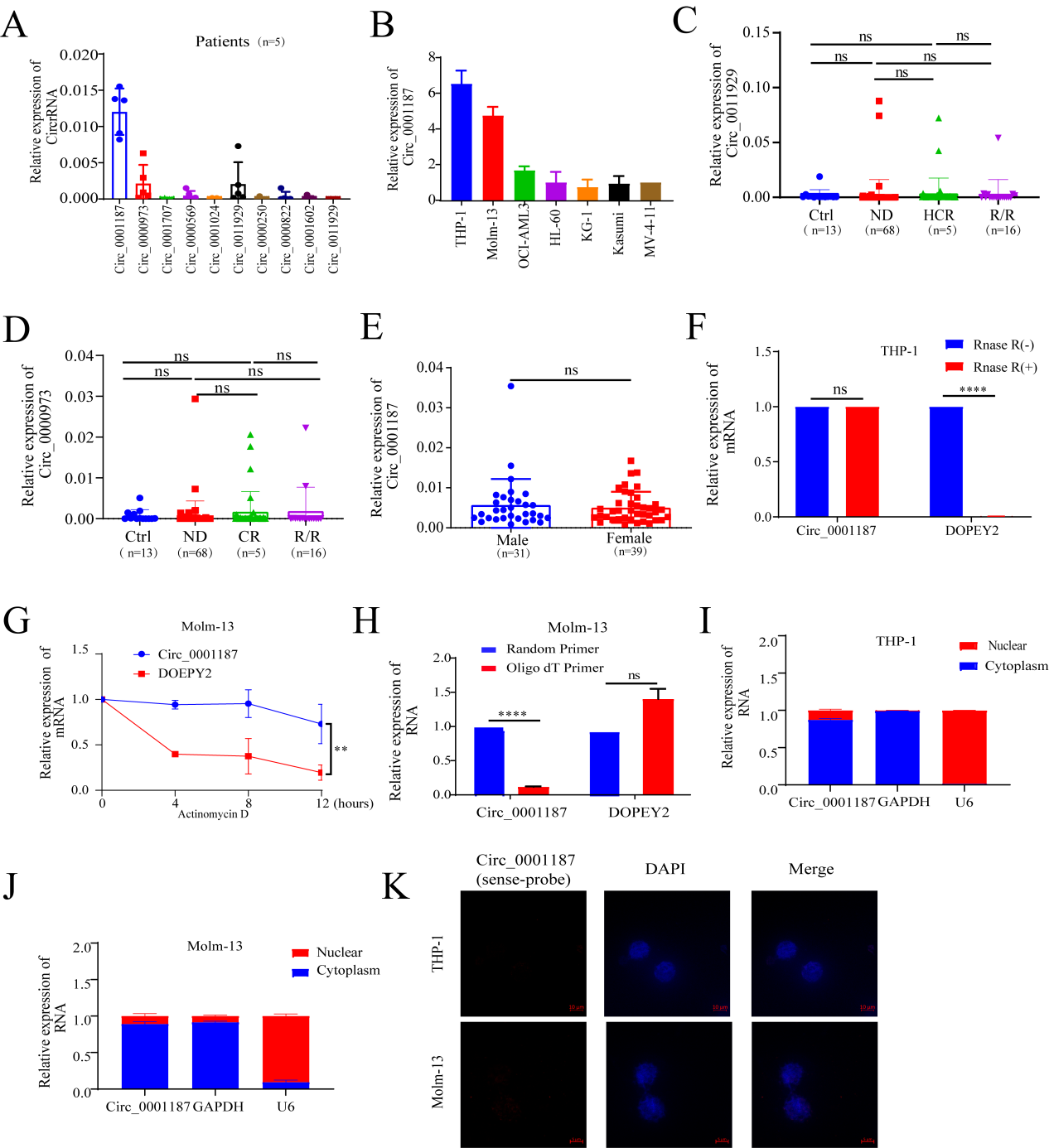
**

**Supplementary Figure 1. Characteristics of Circ_0001187 in AML.** (A) Expression levels of different circRNA in patients. (B) Expression levels of Circ_0001187 in AML cell lines. (C-D) The expressions of Circ_0011929 and Circ_0000973 in AML patients and healthy controls. (E) Relative expression of Circ_0001187 in gender was measured by qRT-PCR. (F) Expression levels of Circ_0001187 and linear DOPEY2 mRNA in THP-1 cells after being treated with RNase R by qRT-PCR. Data were analyzed using Unpaired t-test. (G) Expression levels of Circ_0001187 and linear DOPEY2 mRNA in Molm-13 AML cells treated with actinomycin D by qRT-PCR. (H) The qRT-PCR expression results of Circ_0001187 and DOPEY2 using random primer and Oligo dT primer in Molm-13 AML cells. (I-J) The qRT-PCR analysis of nuclear and cytoplasmic fractionation extracts in THP-1 and Molm-13 AML cells. (K) The results of RNA FISH by using sense probe in THP-1 and Molm-13 AML cells. ***p* < 0.01; *****p* < 0.0001; ns: Not significant.

**Supplementary Figure 2**

**
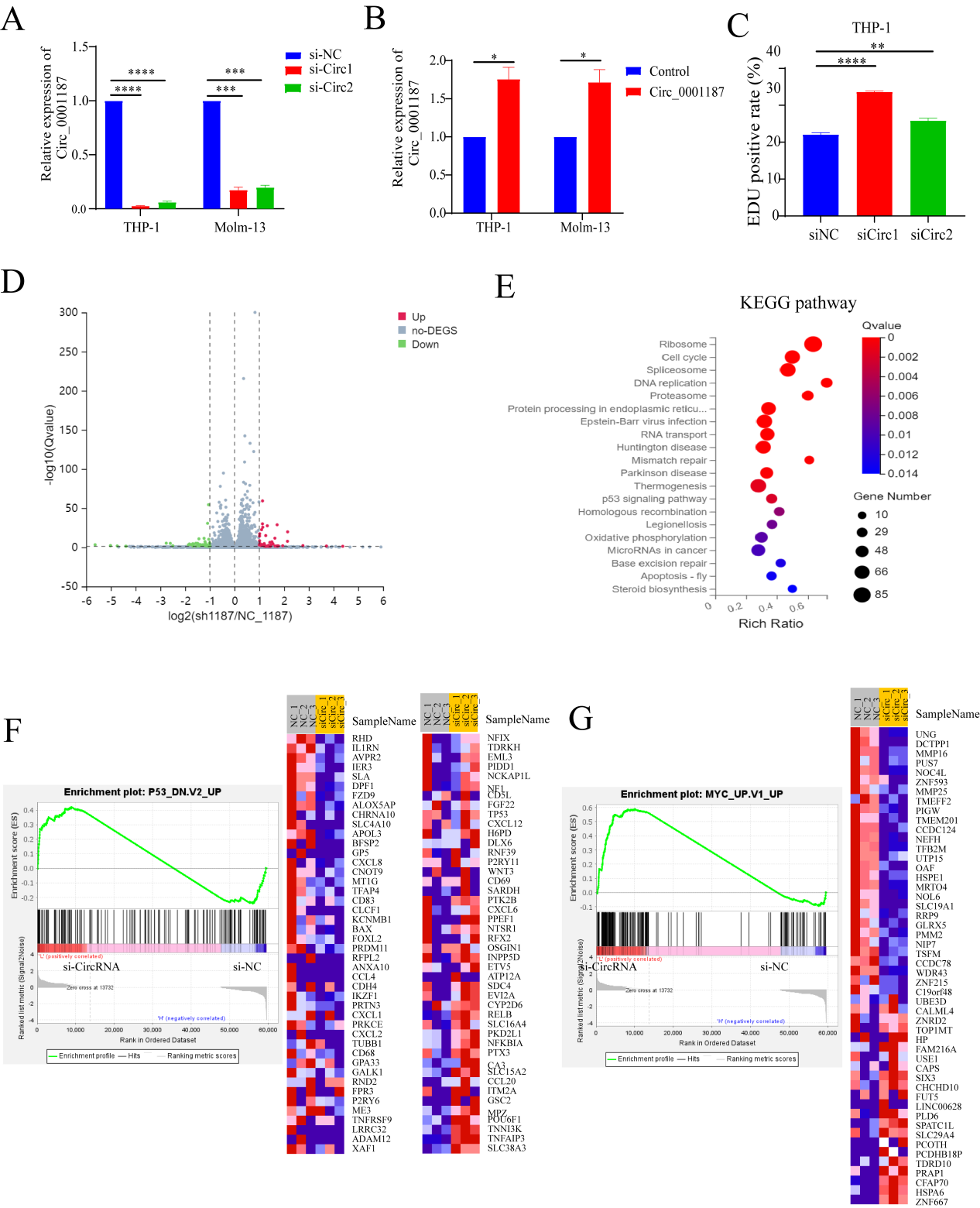
**

**Supplementary Figure 2. Knockdown Circ_0001187 promoted AML progression.** (A-B) The qRT-PCR results of Circ_0001187 in THP-1 and Molm-13 cells with Circ_0001187 knockdown or overexpression. (C) The proliferation results of THP-1 cells transfected si-Circ1 or si-Circ 2 by EDU staining. (D) The expression level of differential genes by RNA-seq analysis. (E) KEGG pathways in Circ_0001187 knockdown compared to the control group. (F-G) GSEA analysis for Circ_0001187 knockdown compared to the control group. **p* < 0.05; ***p* < 0.01; ****p* < 0.001; *****p* < 0.0001.

**Supplementary figure 3**

**
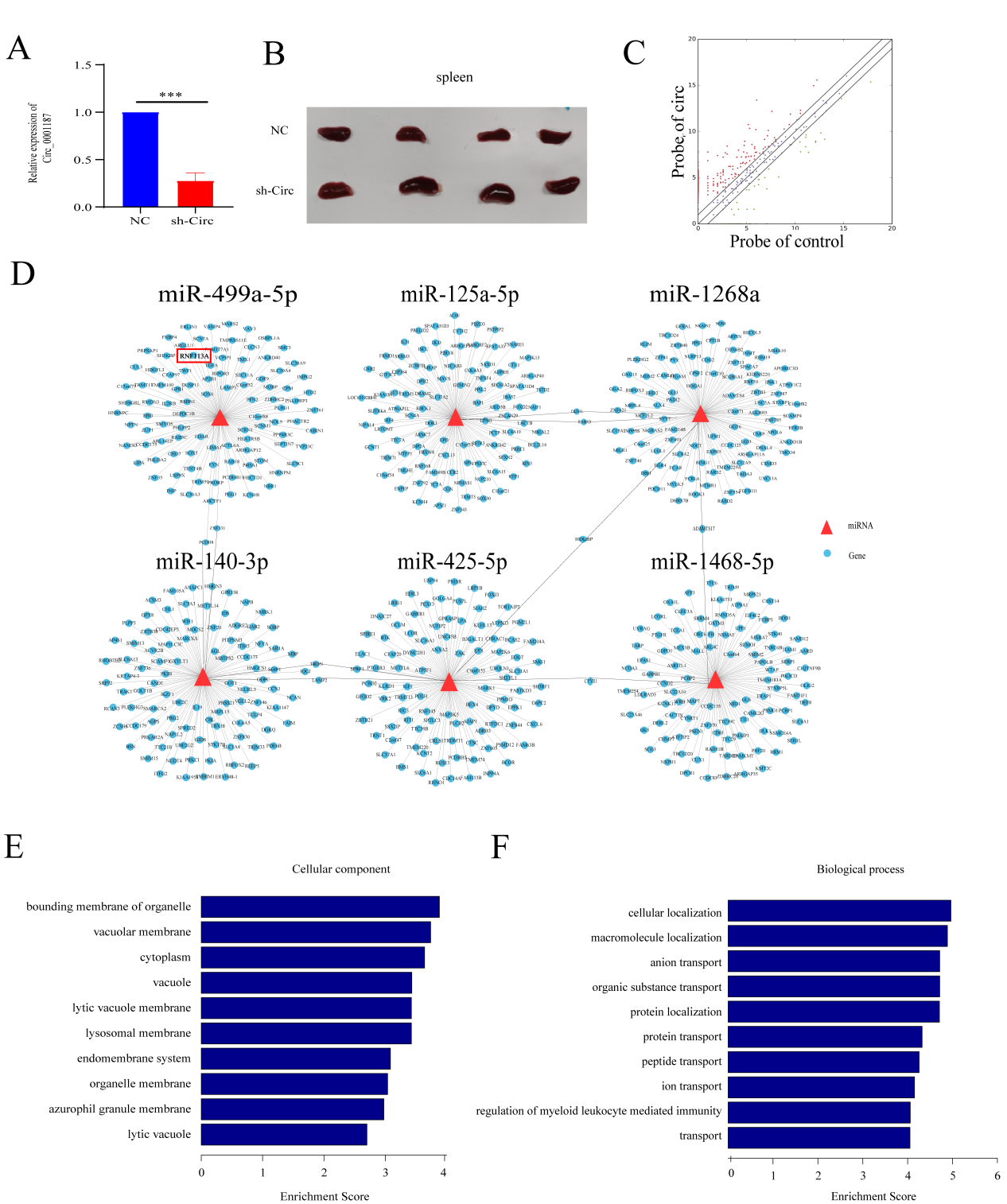
**

**Supplementary figure 3. The top 6 miRNA and its target mRNA prediction network of Circ_0001187.** (A) The qRT-PCR results of Circ_0001187 in mice treated with sh-Circ compared with negative control. (B) The spleen weight of AML mice injected with THP-1 cells transfected with sh-Circ_0001187-GFP or Ctrl-GFP. (C) Differential expression of miRNA from RNA pull-down. (D) miRNA and target mRNA prediction network of Circ_0001187 via RNA pulldown assay. (E-F) The results of GO enrichment correspond to miRNA/mRNA. **p* < 0.05; ****p* < 0.001.

**Supplementary Figure 4**

**
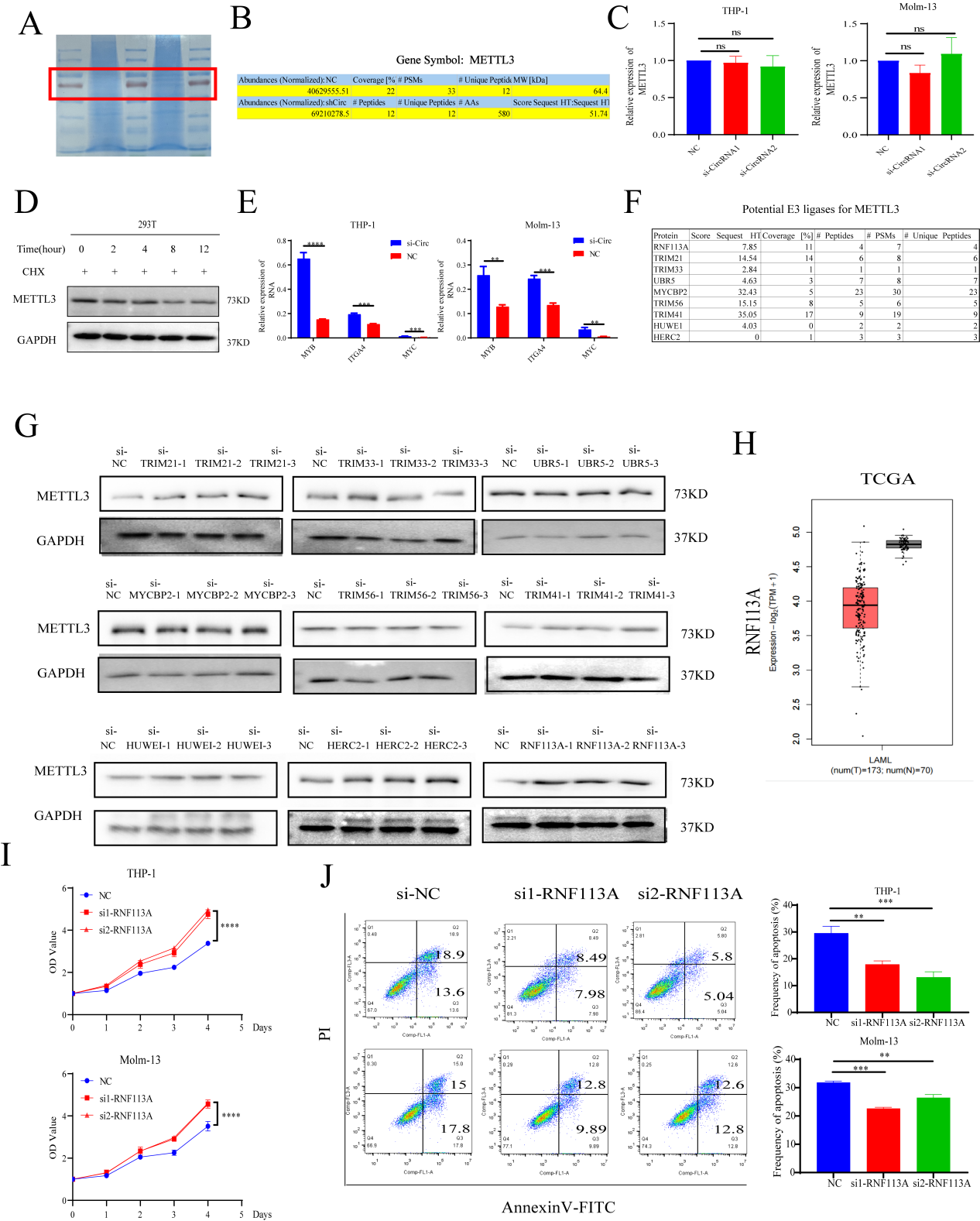
**

**Supplementary Figure 4.** **RNF113A is the E3 ligase of METTL3 in AML.** (A) The results of coomassie blue staining in THP-1with Circ_0001187 knockdown. (B) The protein METTL3 identified by mass spectrometry analysis. (C) The qRT-PCR results of METTL3 in THP-1 and Molm-13 AML cells with Circ_0001187 knockdown. (D) Western blot results of METTL3 in 293-T cells treated with 20 μg/ml CHX at different times. (E) The mRNA leve of MYB, MYC and ITGA4 in THP-1 and Molm-13 AML cells with Circ_0001187 knockdown. (F) The potential METTL3 E3 ligases identified by affinity MS. (G) Western blot results of METTL3 in THP-1 cells transduced with the siRNA of potential E3 ligases respectively compared with negative control. (H) The expression levels of RNF113A from TCGA database. (I) The proliferation results of THP-1 and Molm-13 cells with RNF113A knockdown by CCK-8 assays. (J) The effect of RNF113A knockdown on the apoptosis of THP-1 and Molm-13 cells by flow cytometry. **p* < 0.05; ***p* < 0.01; ****p* < 0.001; *****p* < 0.0001; ns: Not significant.

**Supplementary Figure 5**

**
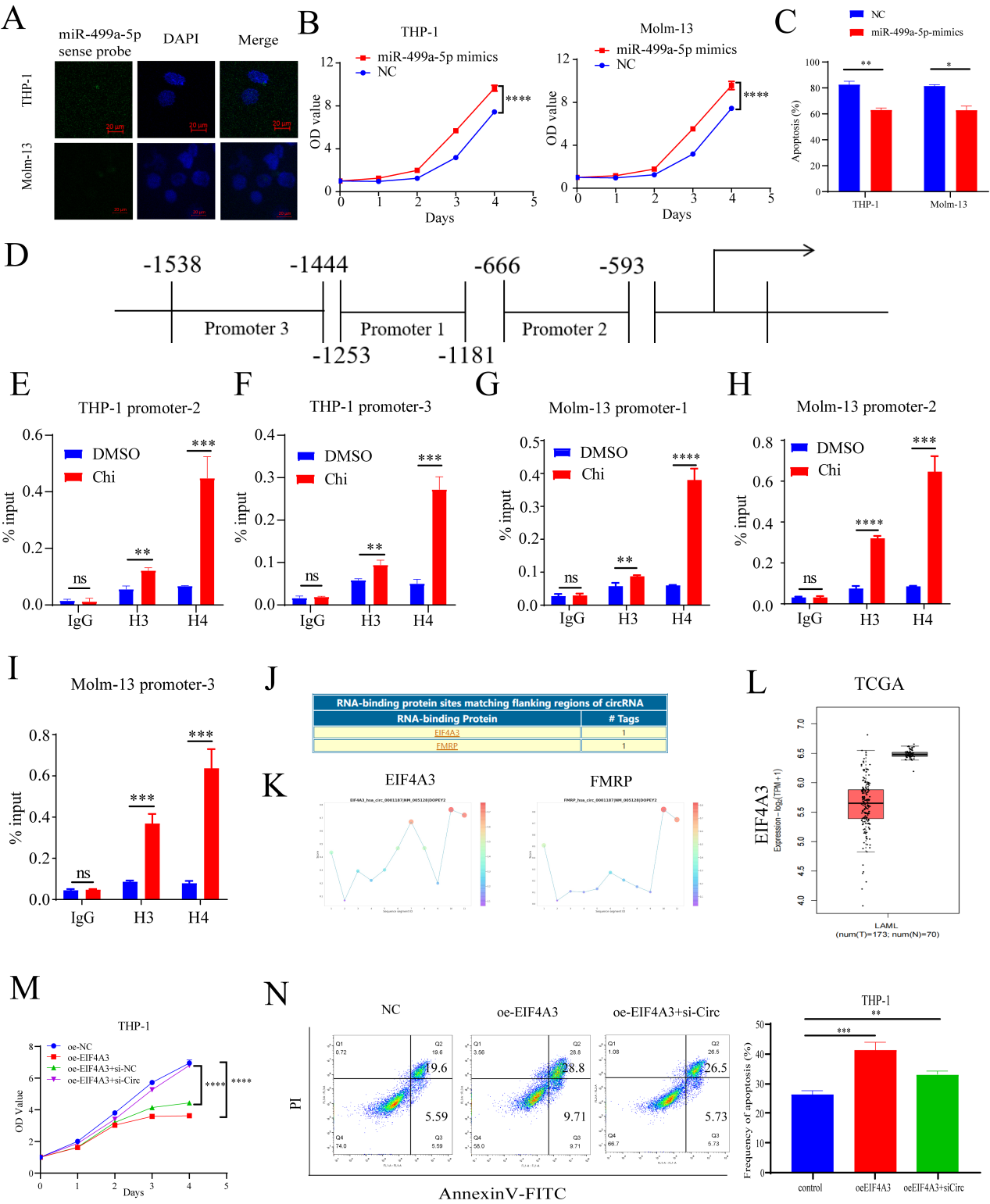
**

**Supplementary Figure 5. The** **function of miR-499a-5p and EIF4A3 in AML.** (A) The results of RNA FISH by using sense probe in THP-1 and Molm-13 AML cells. (B) The effect of miR-499a-5p mimics on the proliferation of THP-1 and Molm-13 cells. (C) The effect of miR-499a-5p mimics on the apoptosis of THP-1 and Molm-13 cells. (D-I) ChIP‒qPCR showing the effect of chidamide on the histone acetylation levels of Circ_0001187 by promoters in THP-1 and Molm-13 cells. (J) The RNA-bing protein sites matching flanking regions of circRNA from Circintercome database. (K) The results from RBPsuit database. (L) The expression levels of EIF4A3 from TCGA database. (M) The effect of oe-EIF4A3 or oe-EIF4A3/siCirc on the proliferation of THP-1 cells. (N) The effect of oe-EIF4A3 or oe-EIF4A3/siCirc on the apoptosis of THP-1 cells. **p* < 0.05; ***p* < 0.01; ****p* < 0.001; ****p < 0.0001; ns: Not significant.

**Supplementary Table 1. Risk classification standard**

| **Risk Category** | **Genetic Abnormality** |
| --- | --- |
| Favorable risk | t (8;21) (q22;q22.1); RUNX1-RUNX1T1 inv(16)(p13.1q22) or t(16;16)(p13.1;q22); CBFB-MYH11 Biallelic mutated CEBPA  Mutated NPM1 without FLT3-ITD or with FLT3-ITD^low^ |
| Poor risk | t(6;9)(p23;q34.1); DEK-NUP214; t(v;11q23.3); KMT2A rearranged; t(9;22)(q34.1;q11.2); BCR-ABL1; inv(3)(q21.3q26.2) or t(3;3)(q21.3q26.2); GATA2; MECOM(EVI1); -5 or del(5q); -7; -  17/abn(17p) ; Complex karyotype; monosomal karyotype; Wild-type NPM1 and FLT3-ITD^high^; Mutated RUNX1; Mutated ASXL1; Mutated TP53 |

**Referance: Acute Myeloid Leukemia, Version 3.2019, NCCN Clinical Practice Guidelines in Oncology**

**Supplementary Table 2. Primers used for quantitative reverse transcription PCR.**

**
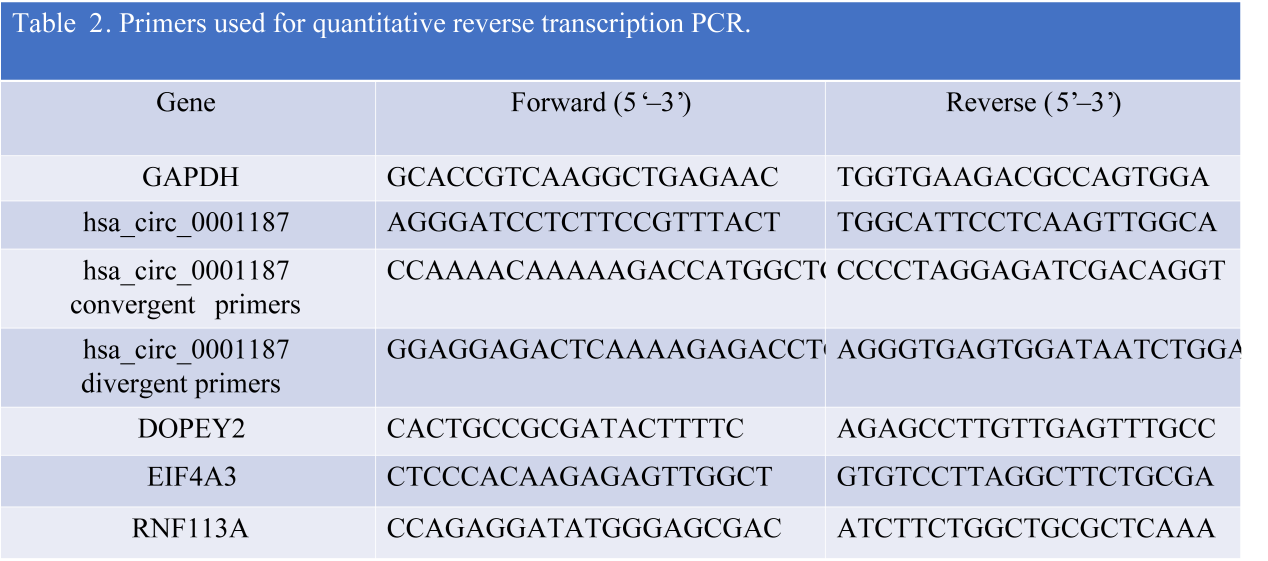
**

**Supplementary Table 3. The sequences for oligonucleotide transfection.**

**
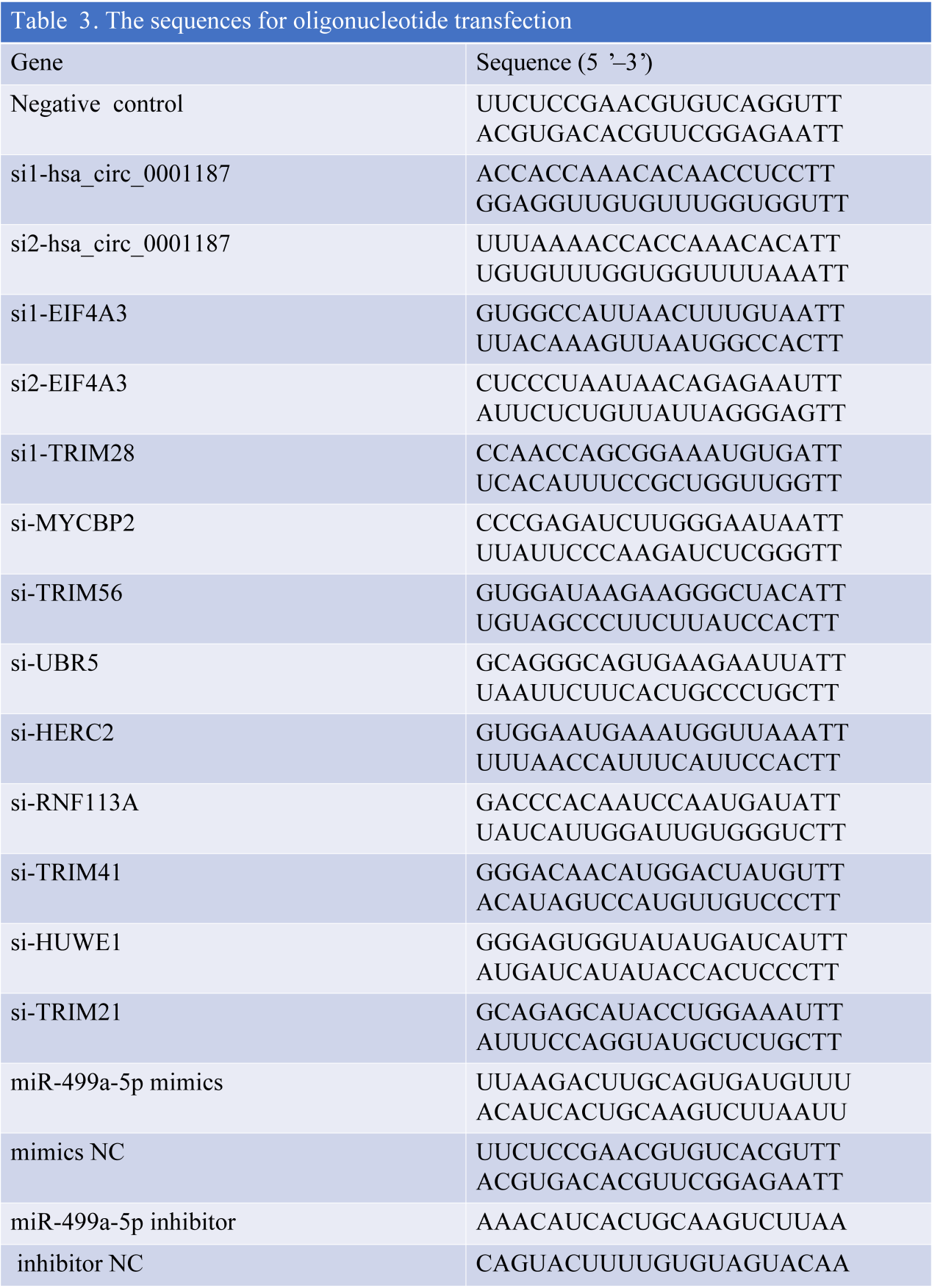
**

**Supplementary Table 4. Primer sets for MSP and CHIP.**

**
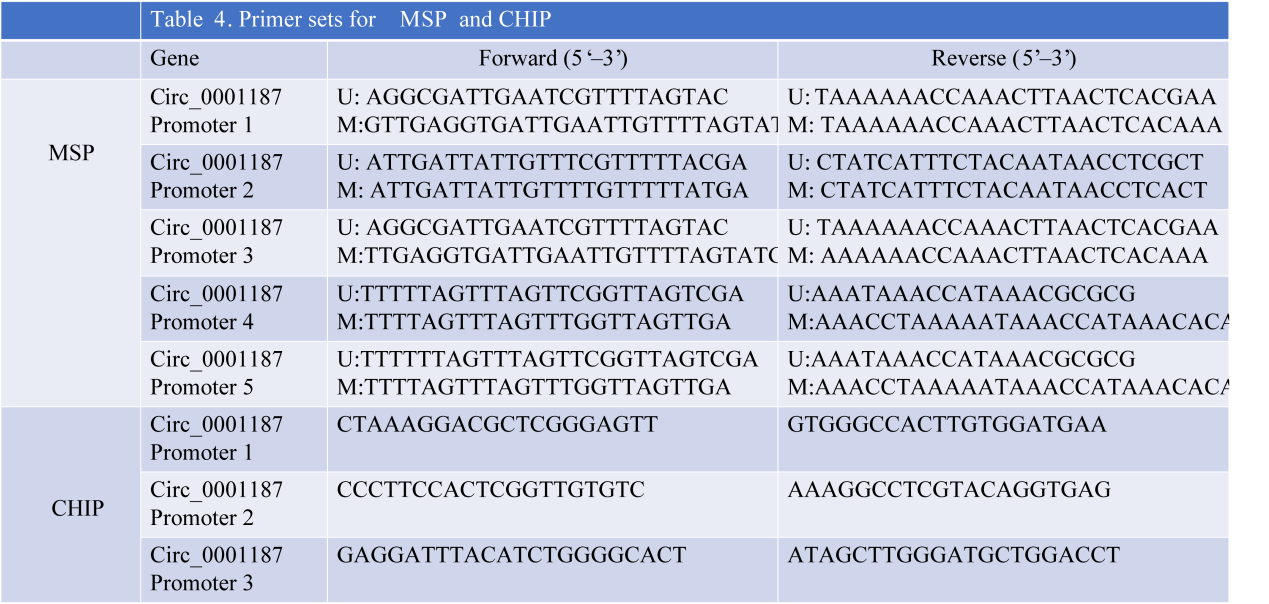
**
